# Design and evaluation of bi-functional iron chelators for protection of dopaminergic neurons from toxicants

**DOI:** 10.1007/s00204-020-02826-y

**Published:** 2020-06-30

**Authors:** Simon Gutbier, Sotiris Kyriakou, Stefan Schildknecht, Anna-Katharina Ückert, Markus Brüll, Frank Lewis, David Dickens, Liam Pearson, Joanna L. Elson, Sylvia Michel, Véronique Hubscher-Bruder, Jeremy Brandel, David Tetard, Marcel Leist, Ilse S. Pienaar

**Affiliations:** 1 In Vitro Toxicology and Biomedicine, Department Inaugurated By the Doerenkamp-Zbinden Foundation, University of Konstanz, 78457 Konstanz, Germany; 2 Faculty of Health and Life Sciences, Northumbria University, Newcastle upon Tyne, NE1 8ST UK; 3 Université de Strasbourg, IPHC, 25 Rue Becquerel, 67087 Strasbourg, France; 4 UMR7178, CNRS, 67087 Strasbourg, France; 5 Department of Molecular and Clinical Pharmacology, University of Liverpool, Liverpool, UK; 6 Institute of Genetic Medicine, Newcastle University, Newcastle upon Tyne, NE1 3BZ UK; 7 School of Life Sciences, University of Sussex, Falmer, BN1 9PH UK; 8 Roche Pharma Research and Early Development, Roche Innovation Center Basel, Grenzacherstrasse, 4070 Basel, Switzerland; 9 Department of Biology, University of Konstanz, Universitaetsstrasse 10, 78464 Konstanz, Germany

**Keywords:** Blood–brain barrier, Dopaminergic neurons, Drug design, Hydroxypyridinones, Iron chelators, LAT1, Parkinson’s disease

## Abstract

While the etiology of non-familial Parkinson’s disease (PD) remains unclear, there is evidence that increased levels of tissue iron may be a contributing factor. Moreover, exposure to some environmental toxicants is considered an additional risk factor. Therefore, brain-targeted iron chelators are of interest as antidotes for poisoning with dopaminergic toxicants, and as potential treatment of PD. We, therefore, designed a series of small molecules with high affinity for ferric iron and containing structural elements to allow their transport to the brain via the neutral amino acid transporter, LAT1 (SLC7A5). Five candidate molecules were synthesized and initially characterized for protection from ferroptosis in human neurons. The promising hydroxypyridinone SK4 was characterized further. Selective iron chelation within the physiological range of pH values and uptake by LAT1 were confirmed. Concentrations of 10–20 µM blocked neurite loss and cell demise triggered by the parkinsonian neurotoxicants, methyl-phenyl-pyridinium (MPP^+^) and 6-hydroxydopamine (6-OHDA) in human dopaminergic neuronal cultures (LUHMES cells). Rescue was also observed when chelators were given after the toxicant. SK4 derivatives that either lacked LAT1 affinity or had reduced iron chelation potency showed altered activity in our assay panel, as expected. Thus, an iron chelator was developed that revealed neuroprotective properties, as assessed in several models. The data strongly support the role of iron in dopaminergic neurotoxicity and suggests further exploration of the proposed design strategy for improving brain iron chelation.

**Electronic supplementary material:**

The online version of this article (10.1007/s00204-020-02826-y) contains supplementary material, which is available to authorized users.

## Introduction

Parkinson’s disease (PD) is the second most common neurodegenerative disorder after Alzheimer’s disease (AD), affecting 1–2% of the population over 65 years of age (de Rijk et al. 2000). It has been estimated that the number of PD cases will double by 2030 (Dorsey et al. 2007), making identification and development of therapeutic agents to prevent, halt or slow down the processes associated with PD neuropathology an urgent aim. The disease is primarily caused by the loss of dopaminergic neurons in the Substantia nigra pars compacta (SNpc) (Lees 2009). Although the exact etiology of neuronal loss is unclear, several contributing factors have been suggested, including intracellular deposition of abnormally aggregated alpha-synuclein protein (α-SYN) that forms a major constituent of so-called ‘Lewy bodies’ (Schildknecht et al. 2013). In association with pathological α-SYN, both impaired proteostasis and mitochondrial dysfunction have been deemed critical drivers of PD pathogenesis (Malkus et al. 2009; Vigouroux et al. 2004; Zabel et al. 2010). Toxicants like methyl-phenyl-tetrahydropyridine (MPTP) can cause human pathology that highly resembles PD (Schildknecht et al. 2017). The history of accidental poisoning with MPTP, which is a contaminant of some illicit drug preparations, has been extensively documented (William Langston 1985). Several environmental agents, such as maneb, dieldrin, paraquat or tebufenpyrad have been examined for potential roles in PD pathology. For some of these pesticides, in particular for the piscicide rotenone, a statistically significant correlation of exposure and disease has been found both in epidemiological studies and in animal experiments (Greenamyre and Hastings 2004; Terron et al. 2018).

Following the initial observation by Lhermitte and others (Lhermitte et al. 1924) of abnormal accumulation of the redox-active biometal, iron within the basal ganglia of PD-affected post-mortem brains, the question arose whether disrupted iron metabolism is either adaptive or disease promoting (Kaur and Andersen 2004). As iron promotes the generation of highly aggressive free radicals via the Haber–Weiss cycle/Fenton reaction (Schildknecht et al. 2013), abnormal accumulation of redox-active metal in the brain could play a central role in PD neuropathology. In this regard, it was suggested that oxidation of iron to the ferric state may drive a vicious circle between excessive pathological levels of reactive oxygen species (ROS), and the intracellular deposition of aggregated α-SYN (Febbraro et al. 2012; Levin et al. 2009; Schildknecht et al. 2013).

The finding that iron accumulates in SNpc-located dopaminergic neurons during PD progression has prompted studies for testing whether iron chelation may be capable of modifying PD progression (Devos et al. 2014; Dusek et al. 2016). Recently, a pilot clinical trial assessed the orally available 3-hydroxy-4(1H)-pyridinone (3,4-HOPO)-based iron chelator, deferiprone (DFP) (Devos et al. 2014). Early-stage PD patients treated for six months showed decreased SNpc iron content, which associated with slowed disease progression, indicated by motor scores. In a follow-up study, the authors reported that 6–12 months of DFP treatment resulted in reduced SN iron levels and also improved Unified Parkinson Disease Rating Scale (UPDRS) scores in early-stage PD patients (Grolez et al. 2015). The authors further reported that PD patients with low serum activity levels of ceruloplasmin, a ferroxidase enzyme important for iron metabolism, responded better to iron chelation therapy. In other work, Martin-Bastida and colleagues (Martin-Bastida et al. 2017) found that DFP has low efficacy for removing iron from the SNpc. The authors further reported only moderate (non-significant) motor symptom improvements in PD patients treated with 30 mg/kg deferiprone. The conflicting results reported in published literature regarding the clinical efficacy of iron chelators against PD-related neurodegeneration calls for further preclinical studies using robust models of the disease to validate and assess the mechanisms of neuroprotection. Moreover, it calls for efforts to explore other, possibly more effective, iron chelators.

The therapeutic efficacy of iron chelators against neurodegenerative disease may be further improved by promoting their uptake into the brain and by altering their affinity pattern for various relevant ions. With this in mind, we explored the anti-PD therapeutic potential of novel hydroxypyridinones (HOPOs) and related structures (Chaves et al. 2018; Workman et al. 2015). The compounds, termed SK1-SK5, were synthesized and assessed for their ability to attenuate dopaminergic neuronal death. As an in vitro screening system for assessing the neuroprotective capacity of the compounds, LUHMES cells were used. These human-derived mesencephalic neural precursor cells can be differentiated into post-mitotic dopaminergic neurons (Schildknecht et al. 2009; Scholz et al. 2011). Such cells have been frequently used in combination with PD-related toxicants to model PD neuropathology, including neuronal loss and intracytoplasmic protein aggregates (Devos et al. 2014; Efremova et al. 2015; Gutbier et al. 2018b; Harris et al. 2018; Hollerhage et al. 2017; Poltl et al. 2012; Schildknecht et al. 2009; Scholz et al. 2011).

The SK compounds were designed to be translocated across the blood–brain barrier (BBB) in a similar way as the PD drug levodopa, which is transported by the large amino acid transporter (LAT1), encoded by SLC7A5 genes (Dickens et al. 2017). As this carrier is known to accept aromatic amino acids, an amino acid side chain was added to the heterocyclic core structure (Singh and Ecker 2018). The rationale for this design feature was to allow brain penetration in high enough concentrations to achieve an anti-parkinsonian therapeutic effect, and thereby avoid excessive metabolism or side effects in the body periphery. Our screen revealed strong neuroprotective properties for one novel chelator in particular, the 3-hydroxy-4(1H)-pyridinone termed SK4, which validates abnormal levels of iron ions as a target for the development of novel therapeutic approaches for slowing PD-related neurodegeneration.

## Materials and methods

### Chemicals

Dibutyryl-cAMP (cAMP), fibronectin, Hoechst bisbenzimide H-33342, resazurin sodium salt, tetracycline and the neurotoxicants 1-methyl-4-phenylpyridinium (MPP^+^) and 6-hydroxydopamine (6-OHDA) were purchased from Sigma-Aldrich (Steinheim, Germany). Recombinant human Fibroblast Growth Factor 2 (FGF-2) and recombinant human glial-derived neurotrophic factor (GDNF) were purchased from R&D Systems (Minneapolis, USA). Erastin was purchased from Selleckchem (Munich, Germany). Chemicals for the synthesis of the iron chelator and control compounds were purchased from either Sigma-Aldrich (Gillingham, UK), Acros Organics (Geel, Belgium) or Fluorochem (Hadfield, UK) and used without further purification, unless otherwise stated.

### Synthesis of novel iron chelators

The synthesis and chemical characterization of precursor 1 (P1: (2L)-3-amino-2-([(tert-butoxy)carbonyl]amino)propanoic acid) and precursor 2 (P2: 3-(benzyloxy)-2-methyl-1,4-dihydropyridin-4-one) was exactly as described in the literature, and is detailed in the supplementary methods and supplementary figure S1. Detailed synthesis protocols and chemical characterization of the SK compounds are found in the supplementary methods and supplementary figures S2–5. Exemplary data are provided here on SK4 ((2L)-amino-3-(3-hydroxy-2-methyl-4-oxo-1,4-dihydropyridin-1-yl)propanoic acid): First 4.9 g (22.66 mmol) of P2 was mixed with 2.9 g (14.2 mmol) of P1, and dissolved in water (100 ml) plus ethanol (100 ml) containing sodium hydroxide (2 g, 50 mmol). The resulting solution was mixed at room temperature (RT) for 8 days continuously. The solution was then acidified to pH 2.0 by the addition of concentrated hydrochloric acid (HCl). Excess solvents were removed under reduced pressure. The resulting residue was mixed with hydrobromic acid (48% w/v, 20 ml) and refluxed for 20 min. The solution mixture was concentrated under reduced pressure, and then the resulting solid was dissolved in water (20 ml), followed by treatment with charcoal and basified (pH 5.0) by adding ammonium hydroxide solution (ammonia water). The resulting solution was cooled to 5 °C for 72 h, after which the brown crystals were precipitated. The crystals were collected, washed with water and dried, rendering the titled compound to become pale brown crystals (3.23 g, 14.27 mmol, 63%). Melting point (Mp): 165–168 oC. ^1^H-NMR (400 MHz, D_2_O/CF_3_COOD) δ_H_ = 1.90 (s, 3H), 3.85 (t, *J* = 7.2 Hz, 1H), 4.07–4.12 (m, 1H), 4.27–4.33 (m, 1H), 6.44 (d, *J* = 8.0 Hz, 1H), 7.36 (d, *J* = 8.0 Hz, 1H); ^13^C-NMR (100 MHz, D_2_O/CF_3_COOD) δ_C_ = 13.4, 49.17, 52.24, 119.54, 141.59, 143.28, 147.33, 170.96, 176.7. High-resolution mass spectrometry (MS) using electron spray ionization gave a measured weight (including 1 extra proton (M + H)) [M + H] of 213.0866 (theoretical [M + H] is 213.0869 for C_9_H_12_N_2_O_4_).

### Metal binding studies

Metal cation solutions were prepared from their perchlorate salts and their concentrations were determined spectrophotometrically for Fe^3+^ or via colorimetric titrations (for Cu^2+^ and Zn^2+^) with EDTA (Titriplex III, Millipore), in accordance with published protocols (Fish 1988; Skoog and Skoog 2004). The acido-basic properties (pKa) of ligands SK2, SK3 and SK4 and their affinity for metals were determined via potentiometric titrations (Cu^2+^, Zn^2+^) and spectrophotometric titrations versus pH (Fe^3+^). The potentiometric titrations were carried out in water (*I* = 0.1 M NaClO_4_), in accordance with a previously published procedure (Gillet et al. 2017), using a 904 DMS Titrando automatic titrator system (Methrom AG; Herisau, Switzerland) with a 2-ml Dosino 800 burette driven by the TiAmo 2.5 software (Methrom AG; Herisau, Switzerland) and a combined glass electrode (6.0234.100; long life, Methrom AG; Herisau, Switzerland) filled with 0.1-M NaCl. The Fe^3+^ complexes were already fully formed at pH 2 and could, therefore, not be studied via this technique. The potentiometric data were refined with the Hyperquad 2008 program (Gans et al. 1996).

The protonation constants of the ligands SK2, SK3 and SK4, and their stability constants with Fe^3+^, were determined by ultraviolet (UV)–visible spectrophotometric titrations versus pH, by simultaneously recording pH and UV–visible spectra using a Metrohm UV–visible spectrophotometer (Cary 60 model, Agilent). Between pH =  − 0.5 and 2, the batch technique was used; while, classical titrations were carried out for pH values between 2 and 12, following a published procedure (Gillet et al. 2017). The ionic strength was not fixed at pH < 1 in the batch titrations and no decomposition of the ligands was observed, even in strongly acidic conditions.

The spectrophotometric data were fitted with HypSpec software (Gans et al. 1996), accessible at https://www.hyperquad.co.uk, to calculate the protonation constants of the ligands, the stability constants (log ß) of the formed species and the co-ordination model of the studied systems. The data for Fe^3+^, Cu^2+^ and Zn^2+^ hydrated species and their solubility products were taken into account in the equilibrium model (Baes and Mesmer 1986).

### Cell culture

LUHMES cells have a normal human genome, a dopaminergic phenotype, neurotypical electrophysiological properties and sensitivity to PD-related neurotoxicants, including rotenone, 1-methyl-4-phenyl-1, 2, 3, 6-tetrahydropyridine (MPTP) and its metabolite MPP^+^. Handling of LUHMES cell cultures was performed as previously described (Gutbier et al. 2018a; Scholz et al. 2011). Briefly, the conditionally immortalized cells were maintained in proliferation medium (PM) that consisted of advanced Dulbecco's Modified Eagle Medium (DMEM)/F12, 2 mM l-glutamine, 1xN2 supplement (Invitrogen, CA, USA) and 40-ng/ml FGF-2, and were kept in a 5% CO_2_/95% air atmosphere at 37 °C. Cells were passaged every other day. For differentiation, 8 million cells were seeded in a Nunclon T175 tissue culture flask pre-coated with 50-µg/ml poly- l -ornithine (PLO) and 1-µg/ml fibronectin in water. After 24 h, the medium was changed to differentiation medium (DM), consisting of advanced DMEM/F12 supplemented with 2-mM l -glutamine, 1 × N2, 2.25-µM tetracycline, 1-mM dibutyryl 3′,5′-cyclic adenosine monophosphate (cAMP) and 2-ng/ml recombinant human GDNF. After 48 h, cells were trypsinised and seeded at a density of 1.5 × 10^5^ cells/cm^2^ into dishes pre-coated with 50-µg/ml PLO and 1 µg/ml fibronectin in DM.

### In vitro neurotoxicity models and compound treatment

LUHMES cells, differentiated into mature, post-mitotic dopaminergic neurons, were treated with one of the four neurotoxicants, to create well-characterized in vitro models of PD. Prior to neurotoxicant exposure, cells were treated with either an SK or control compound for 1 h before addition of the neurotoxicant. All compounds (using a stock concentration of 50 mM) were easily dissolvable in distilled H_2_O (dH_2_O) without requiring heating. The only exception was SK2, where the stock concentration was reduced to 5 mM, due to poor water solubility of the compound. SK and control compounds were applied to the cells at concentrations which ranged from 12.5 to 500 µM under standard incubator conditions (37 °C in a humidified 95% air, 5% CO_2_ atmosphere).

Following the preincubation period with a compound of interest, cells were treated with the parkinsonian mimetic, MPP^+^, the active metabolite of MPTP, which induces dopaminergic neuronal toxicity by inhibiting mitochondrial complex I activity (Dauer and Przedborski 2003). MPP^+^ (5 µM, Sigma), dissolved in dH_2_O, was applied to the cells for 72 h before further assessments.

Cells were also treated with the neurotoxicant 6-OHDA, which is relatively selective for uptake by dopaminergic neurons due to the high affinity such neurons’ plasma membrane transporters hold for this molecule (Luthman et al. 1989). In this neurotoxicant-induced model of cell death, neurodegeneration occurs principally by means of excessive generation of ROS, to ultimately evoke oxidative stress-related cytotoxicity (Blum et al. 2001). After the compound preincubation period, cells were treated with 100-µM 6-OHDA (Sigma-Aldrich), dissolved in dH_2_O. Cells were toxin-treated for 18 h, before further measures were taken.

Ferroptosis, a non-apoptotic form of iron-dependent cell death that occurs due to lethally high levels of lipid hydroperoxides (Stockwell et al. 2017) was initiated via application of the ferroptosis activator, erastin (Eradicator of RAS and Small T antigen-expressing cells). Similar to the other toxin cellular conditions, either an SK or a control compound was pre-applied to the cells for 1 h before exposure to erastin (which lasted 24 h). Erastin was applied to the cells at a concentration of 1.25 µM [10-mM stock in dimethyl sulfoxide (DMSO)], prediluted to 1:800, with the final concentration applied to the cells that was 1:10.

Finally, iron-mediated cell damage was initiated by the addition of ferrous sulfate. The cells were pretreated with an SK compound for 1 h. Fe^2+^SO_4_ was then added for a period of 4 days, after which viability was assessed via the Resazurin reduction assay. Neuronal morphology was also determined by staining the cells with an anti β-III-tubulin antibody (Sigma, rabbit, 1:1000) and then a secondary antibody (1:1000, anti-rabbit IgG Alexa Fluor 555; Invitrogen) for fluorescent (yellow) detection.

### General cell viability endpoints

Resazurin: Metabolic activity was detected by means of a resazurin reduction assay. Briefly, resazurin solution was added to the cell culture medium to obtain a final concentration of 10 µg/ml. After incubating for 30 min at 37 °C, the fluorescence signal was measured at an excitation wavelength of 530 nm, using a 590-nm long-pass filter to record the emission. Fluorescence values were normalized by setting fluorescence values of untreated wells as 100%.

LDH release: In a separate assay, lactate dehydrogenase (LDH) activity was measured in the supernatant and in the corresponding cell homogenates. After the media had been transferred into separate plates, cells were lysed in PBS/0.1% Triton X-100 for 2 h. A total of 20 μl of sample was then added to 180 µl of reaction buffer containing nicotinamide adenine dinucleotide (NADH, 100 μM) and sodium pyruvate (600 μM) in potassium phosphate buffer (pH 7.4). Absorption at 340 nm was measured at 37 °C in 1-min intervals over a period of 15 min. The slope of NADH consumption was calculated. The ratio of LDH_supernatant_/LDH_total_ was calculated using the slopes of supernatant and homogenate. Control data were subtracted from LDH values. Final LDH release data is expressed as percentage of total LDH.

### Neurite area as an indicator of cell viability

Calcein-AM: Labeling of live cells was performed with 1-µM Calcein-AM / 1-µg/ml Hoechst H-33342 for 30 min at 37 °C. Images were collected in two different fluorescent channels using an automated microscope (Array-Scan VTI HCS Reader; Thermo Fisher, PA, USA). Analysis was performed by epifluorescence imaging using an automated microplate-reading microscope (ArrayScan VI HCS Reader, Cellomics, Pittsburgh, PA, USA) equipped with a Hamamatsu ORCA-ER camera (resolution 1024 × 1024; run at 2 × 2 binning). Nuclei were identified as objects using channel 1 (365 ± 50/461 ± 15 nm) and according to their average size, area, shape and intensity. The calcein signal was detected in channel 2 (475 ± 40/525 ± 15 nm). An algorithm quantified all calcein-positive cells as viable and only Hoechst H-33342-positive nuclei as “not viable” cells. For evaluating the neurite areas, nuclei masks were first determined in channel 1, and then expanded and transferred to channel 2. All calcein-positive pixels lying outside of these masks (somatic area) were counted as neurite area.

### Three-Dimensional neuronal organoids

Three-Dimensional (3D) neuronal organoids were generated by differentiating LUHMES cells for two days, as described for LUHMES cell culturing. On day 2 of differentiation, cells were detached and seeded in round-bottom ultra-low attachment 96-well plates (#7007, Corning Costar) at a cell density of 5000 cells/well in 100-µl LUHMES differentiation medium (DM). To facilitate spheroid formation, plates were centrifuged at RT for 5 min at 300 × g. Organoids formed spontaneously from the cell pellets within 48 h. Half medium was changed every 2–3 days. On day 8 of differentiation, LUHMES organoids were plated on Matrigel-coated (1:200) 96-well plates (#83.3924, Sarstedt) to allow neurite outgrowth. After 48 h, plated organoids were treated with the ferroptosis-inducer, erastin for 24 h, with or without 1 h pre-treatment with ferrostatin-1, the established neuroprotective iron chelator desferoxamine (DFO) (Poltl et al. 2012), or SK4, which the initial neuroprotective screen highlighted as a lead compound. Cell viability was determined using calcein-AM (1 µM) staining, while neurite area was determined by means of propidium iodide (1 µg/ml) staining, the staining reagents that were applied for 1 h. Mosaic fluorescent images were then taken of the cells using automated microscopy (Axio Observer A1, Carl Zeiss, Germany), which utilized an objective size 5x/0.15 EC Plan-Neofluor. Twelve images per well were taken and stitched together using Zeiss “Zen” imaging software (version 1.1.2.0, Blue edition). Images were analyzed using ImageJ software (version 1.51 s, Rasband, U.S. National Institutes of Health). Neurite area was calculated as the total neurite area, and cell viability as the relative propidium iodide fluorescence intensity to a positive 100% dead control (0.5% Triton-X100).

For immunostaining, plated spheres were fixed on an 8-well µ-slide (#80,826, Ibidi, Germany) with 4% paraformaldehyde (PFA) for 1 h at RT. After washing with phosphate-buffered saline (PBS), specimens were permeabilized with 0.6% Triton-X100 for 30 min at RT. Subsequently, they were blocked with blocking buffer (5% fetal bovine serum (FBS) and 0.1% Triton-X100 in PBS). Anti-NF200 primary antibody (#N0142, Sigma) was applied diluted in blocking buffer (1:1000) overnight at 4 °C. After washing with PBS, secondary antibody (#A2112, anti-mouse IgG1 Alexa Fluor 555, Invitrogen) was applied diluted in blocking buffer (1:1000) for 1 h at RT. Images were recorded with a Zeiss LSM 880 confocal microscope (40×/1.4 Plan apochromat, oil based) and processed in ImageJ.

### LAT1-mediated uptake and trans-stimulation assay

HEK293 cells, stably expressing pcDNA3.1 V5-6xHis (control) or pcDNA3.1 SLC7A5-V5-6xHis (LAT1), were generated and cultured in DMEM containing 10% FBS (Dickens et al. 2013, 2017). HEK293 control and HEK293 LAT1 cells were plated 24 h before the transport assays were performed. The wells were washed with transport buffer [Hank’s buffered saline solution + 25 mM HEPES + 0.1% w/v bovine serum albumin (BSA) at pH 7.4] pre-warmed to 37 °C. ^3^[H]-phenylalanine [American Radiolabeled Chemicals (ARC), St. Louis, MO, USA] at a radiotracer concentration of 0.15 µCi/ml and unlabelled phenylalanine (ARC), giving a final concentration of 1 µM, was included in the transport buffer. This was added to the cells for 3 min at 37 °C in the absence/presence of 1-mM leucine, SK4 or SK4C2. At the end of the uptake assay the cells were washed 3 × with ice-cold transport buffer. Cell lysis was carried out by adding 5% w/v sodium dodecyl sulfate (SDS) for at least 15 min at 37 °C. Liquid scintillation (LS) counting on the cell lysates was performed with a 1500 Tri Carb LS counter (Packard Instrument Company, Germany). The LAT1-mediated uptake of phenylalanine is determined by subtracting the uptake of HEK293 control cells from HEK293 LAT1 cells. The data values were converted to percentages by normalizing to the average uptake in the vehicle treated cells.

Trans-stimulation is similar to the uptake assay except that the HEK293 LAT1 cells were preloaded for 3 min at 37 °C with transport buffer containing 1-µM ^3^[H]-phenylalanine as a radiotracer. The cells were then washed with pre-warmed transport buffer, after which SK4 (1 mM) was incubated with the cells for 3 min. Under such conditions, external LAT1 substrates are transported into cells, while LAT1 substrates are exported from cells as part of the overall antiporter transport cycle. Cell washing, lysis and scintillation counting were then performed similar to what is described (Dickens et al. 2013, 2017) for performing the uptake assay.

### Electrochemical detection of nitric oxide

Interaction of the SK compounds with nitric oxide (·NO) was detected by the application of a ·NO-sensitive electrode (AmiNO-700, Innovative Instruments, Tampa, FL, USA), as previously described (Schildknecht et al. 2011). The SK compounds (50 µM in 10-mM PBS, pH 7.4,) were incubated with the ·NO-donor Spermine-NONOate (10 µM) (Cayman Chemicals, Ann Arbor, MI, USA). Measurements were performed in glass tubes at 37 °C, and all samples contained 100-µM desferoxamine to exclude any contribution of iron to the observed effects. As the release of ·NO by Spermine-NONOate is temperature dependent, the freshly prepared ice-cold Spermine-NONOate was injected into pre-warmed (37 °C) PBS/compound mixture (990 µl). This procedure leads to an almost linear increase in ·NO release within the first few minutes after injection, followed by a steady-state phase, characterized by a slow but gradual decline in ·NO release in consequence to Spermine-NONOate’s half-life time of ca. 45 min (Maragos et al. 1991). In the present experiment, the initial linear increase was detected. As positive controls, we used xanthine oxidase (1 mU/ml) together with its substrate hypoxanthine (500 µM), a superoxide-generating system.

### Interaction of SK compounds with superoxide or peroxynitrite

With the experiments conducted in 96-well plates, interaction between the SK compounds (50 µM) and superoxide was monitored as the oxidation of dihydroethidium (DHE) (5 µM, Sigma-Aldrich). SK compound interaction with peroxynitrite was monitored by detecting dihydrorhodamine (DHR) 123 (1 µM, Sigma-Aldrich) oxidation in PBS (10 mM). Peroxynitrite was generated by Sin-1 (50 µM), superoxide was generated by xanthine oxidase (1 mU/ml) (Sigma-Aldrich) in combination with its substrate, hypoxanthine (500 µM, LKT Laboratories, St. Paul, MN, USA). Freshly prepared Sin-1 or xanthine oxidase was stored on ice and added to pre-warmed buffer/compound for 20 min at 37 °C. Fluorescence of DHR 123 (*λ*_ex_ = 485 nm, *λ*_em_ = 538 nm) and DHE (*λ*_ex_ = 485 nm, *λ*_em_ = 575 nm) was read using a TECAN Infinite 200 microplate reader (Männedorf, Switzerland).

### Interaction of SK compounds with hydroxyl (·OH) radicals

In 96-well plates, hydroxyl radicals (·OH) were generated by combining FeSO_4_ (20 µM) and H_2_O_2_ (50 µM) in H_2_O. For the readout, desoxyribose (5 mM) was added for the interaction with ·OH to form malondialdehyde. The SK compounds and desferoxamine, were added at the concentrations indicated in the graphical display of the data and incubated for 15 min at RT. The amount of malondialdehyde formed was then detected by the combination of 1/3 volume sample mix, 1/3 volume trichloroacetic acid (2.8 g/100 ml) and 1/3 volume of thiobarbituric acid (1 g in 100 ml of 50 mM NaOH). The mixture was boiled for 20 min at 95 °C. Absorption was then measured at 532 nm.

### Data handling and statistics

If not mentioned otherwise, all data values are expressed as means ± the standard deviation (SD). If not indicated otherwise, experiments were performed at least three times (i.e., using three different cell preparations), with at least three technical replicates per condition. Statistical methods for analyzing the various data sets are indicated directly in the figure legends.

## Results

### Design rational of novel iron chelators

**Fig. 1:**
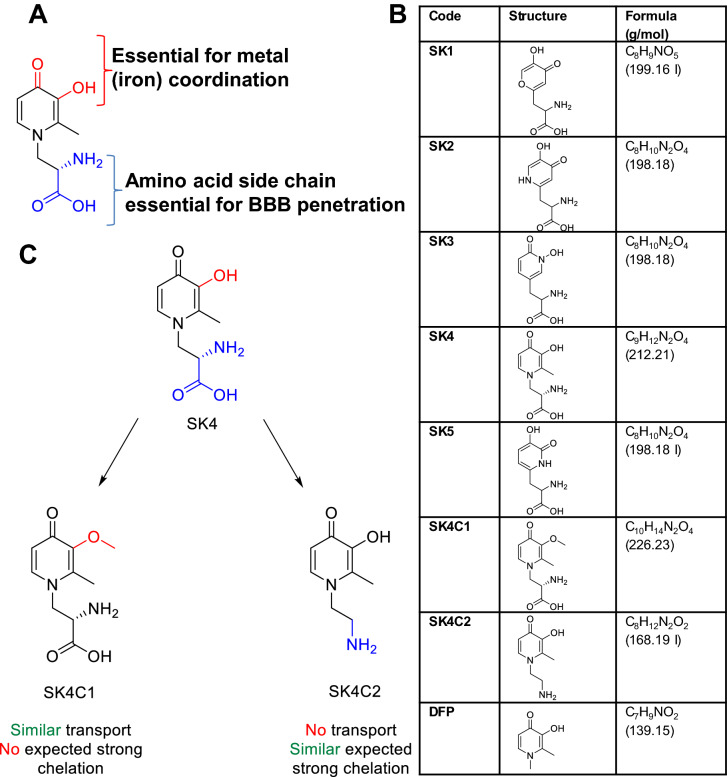
Design hypothesis for novel iron chelators. Iron chelators characterized in this study were designed for both optimized iron co-ordination and enhanced blood brain barrier transport. **a** All compounds have two substructures with different function: (i) a group essential for the iron co-ordination (red) and (ii) the amino acid side chain to facilitate LAT1-mediated uptake into endothelial cells of the BBB (blue). **b** Acronyms, structures and molecular weights of all chelators tested within this study. **c** Design of proof-of-concept control compounds. SK4C1 and SK4C2 are derivatives of SK4 with a functional inactivation of one of the substructures depicted in **a**. SK4C1 has a methoxy group (in place of the hydroxy), which inhibits iron chelation and SK4C2 is a primary amine (not an amino acid), which is not transported by LAT1 (color figure online)

Here, we used the structure of the LAT1 substrate L-DOPA, as well as features of the iron-chelating HOPOs as the basis for designing brain-permeable iron chelators. For this purpose, the aromatic ring of L-DOPA (catechol group) was replaced by a metal chelator moiety, which is similar to DFP (Fig. 1a). We hypothesized that such modified amino acids will remain LAT1 substrates and should have clinically favorable CNS-targeting properties. Further structural variations were introduced to some compounds to change their susceptibility to enzymatic inactivation by methylation or glycosylation. Thus, the compounds SK1-5 were designed and synthesized (Fig. 1a, b, S1-4). In addition, two variants of SK4 were designed and produced to provide analogs lacking chelation properties (SK4C1) or LAT1 affinity (SK4C2) (Fig. 1c; S5).

### Protection of neurons from erastin-induced ferroptosis

**Fig. 2:**
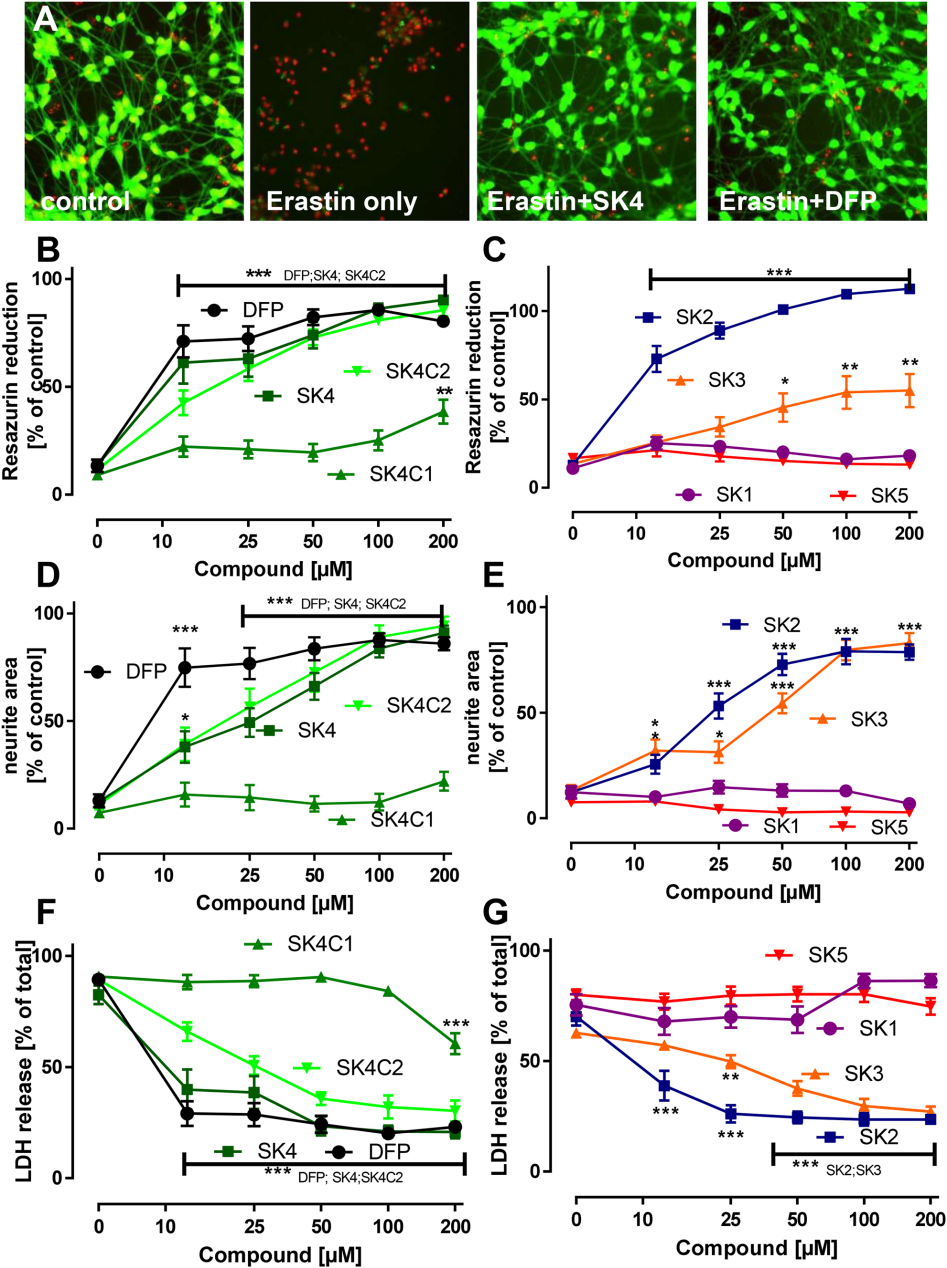
Protection of neurons from erastin-induced ferroptosis by SK compounds. **a** Differentiated LUHMES neurons were treated on day 6 (d6) with erastin (1.25 μM) in the presence or absence of SK4 (200 μM) or deferiprone (DFP; 200 μM). After 24 h, the cells were stained with calcein-AM (green stain of live cells) and H-33342 (red stain for live and dead cells). Representative images are shown. The width of one image corresponds to 300 μm of the original cultures **b**/**c**: Neuroprotection against erastin-induced ferroptosis of LUHMES (d6) cells was tested as in **a**, but resazurin reduction was used as quantitative endpoint. D/E: Experiments were performed as described in **a**–**c**, but neurite integrity was quantified by automated microscopy as alternative endpoint **f**/**g**: Experiments were performed as described in **a**–**e**, but the release of lactate dehydrogenase (LDH) was measured as death endpoint. Data are means + SEM of three independent experiments (color figure online)

We used a cellular model of programmed cell death mediated by endogenous cellular iron for bioactivity profiling. Ferroptosis (Dixon et al. 2012) was triggered by erastin. This model compound was chosen for the initial screen, as it neither affects iron directly, nor is it known to damage any cellular structures directly (Yang et al. 2014). SK2 and SK4 both protected neurons to a similar extent as DFP, as assessed by the test endpoints resazurin reduction, neurite area and LDH release. SK3 was active, but less potent; SK1 and SK5 completely lacked the ability to protect neurons from ferroptosis following erastin exposure (Fig. 2a–g). The modifications of SK4 showed the expected properties; in this regard, SK4C1, which lacks any metal chelation properties, did not protect neurons against erastin toxicity. In addition, SK4C2, which represents the structural modification to disturb LAT1 transport, retained similar in vitro bioactivity as SK4 (Fig. 2a, d, f).

### Biologically relevant features different from iron chelation

**Fig. 3:**
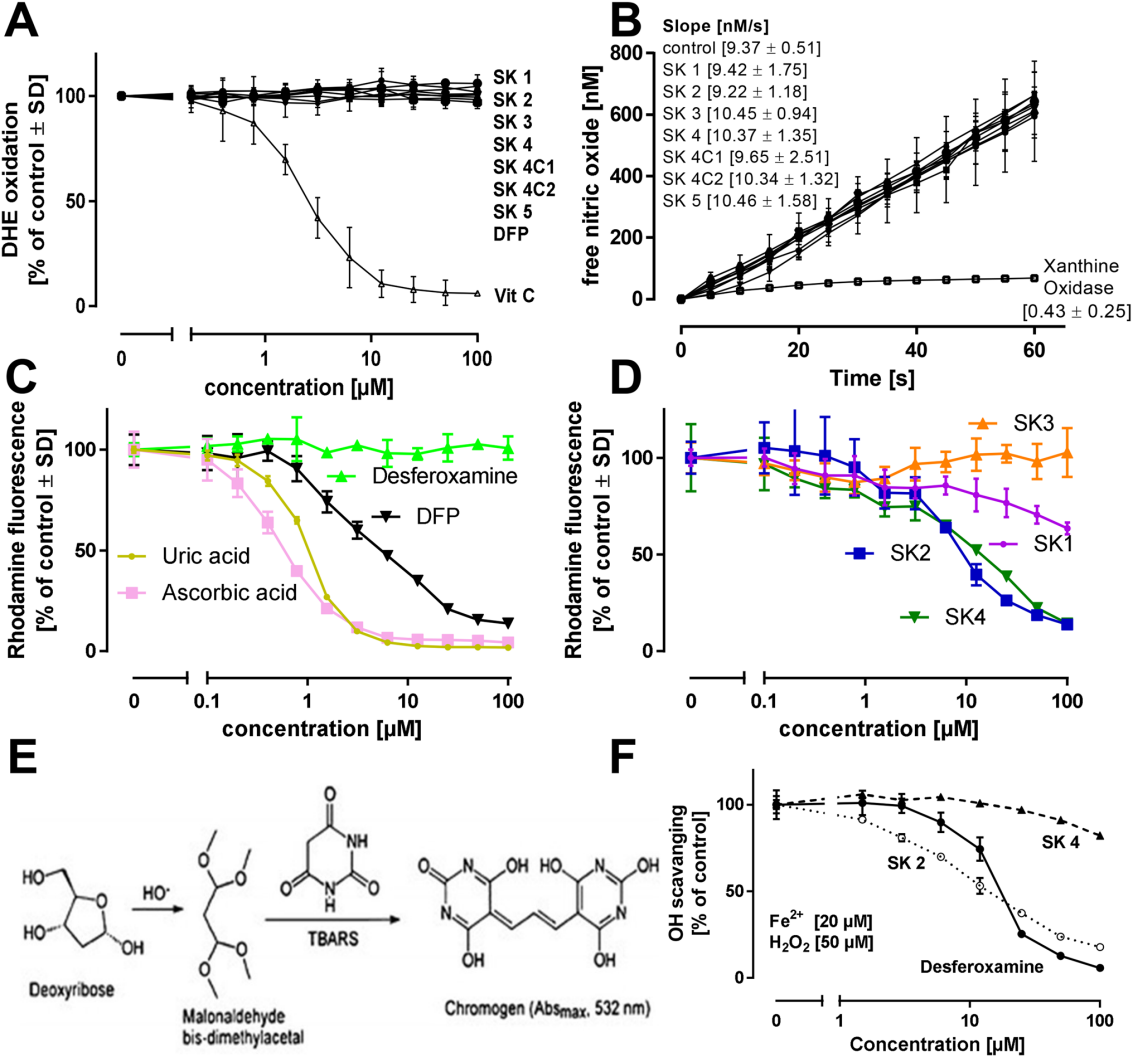
Antioxidant properties of iron chelators. **a** Interaction of SK compounds with superoxide. The SK compounds (50 µM) were incubated with xanthine oxidase (1 mU/ml), hypoxanthine (500 µM), and DHE (5 µM) for 20 min. **b** Interaction of SK compounds (50 µM) with nitric oxide was investigated by their incubation with the ·NO-donor Spermine-NONOate (10 µM) and the detection of free ·NO by a ·NO-selective electrode. As positive control, superoxide was generated by xanthine oxidase/hypoxanthine to quench ·NO. **c** + **d** Interaction of SK compounds with peroxynitrite was investigated by application of the peroxynitrite-generating compound Sin-1 (50 µM). As readout, DHR 123 (1 µM) was added, its oxidation was followed by the detection of Rhodamine fluorescence. Desferoxamine and deferriprone (DFP) were tested as alternative iron chelators, uric acid and ascorbic acid served as positive controls. **e** + **f** Interference of SK compounds with Fe^2+^/H_2_O_2_-derived hydroxyl radical (·OH) generation. Ferrous iron (20 µM) and H_2_O_2_ (50 µM) were combined to allow ·OH generation. As readout, · OH-dependent formation of malondialdehyde was assessed. Data are means + SEM of three independent experiments. Significance tests were not performed for individual data points

The adverse effects of free iron ions in biological systems can be mediated by oxidative stress (Monzani et al. 2019). Therefore, antioxidants often act as false positives in drug discovery programs for identifying effective iron chelators. While antioxidant properties may provide added value to an anti-neurodegenerative drug, one has to be careful during the initial stages of profiling the drug’s properties not to mistake a drug’s protective properties for iron chelation potency. We, therefore, profiled the ability of the SK compounds to directly (independent of iron chelation) scavenge superoxide, nitric oxide, peroxynitrite and hydroxyl radicals. None of the tested compounds reacted with superoxide or nitric oxide (Fig. 3a, b). However, DFP, SK2 and SK4 proved to be peroxynitrite scavengers of similar potency, but clearly less potent than the more well-known scavengers, urate and ascorbate.

Two promising drug candidates, SK2 and SK4, were further profiled in a hydroxyl radical scavenging assay. SK2 was significantly more potent than SK4. Its activity was in a similar range to that of DFO, an iron scavenger known to also have distinct antioxidant properties (Bartesaghi et al. 2004) (Fig. 3a–e). Based on these data, any protection seen with SK2 in a complex bioassay (in vitro or in vivo) may be due either to specific iron chelation or to unspecific radical scavenging. SK4 appeared as a more promising tool compound to test for the specific role of iron in neurodegeneration.

**Fig. 4:**
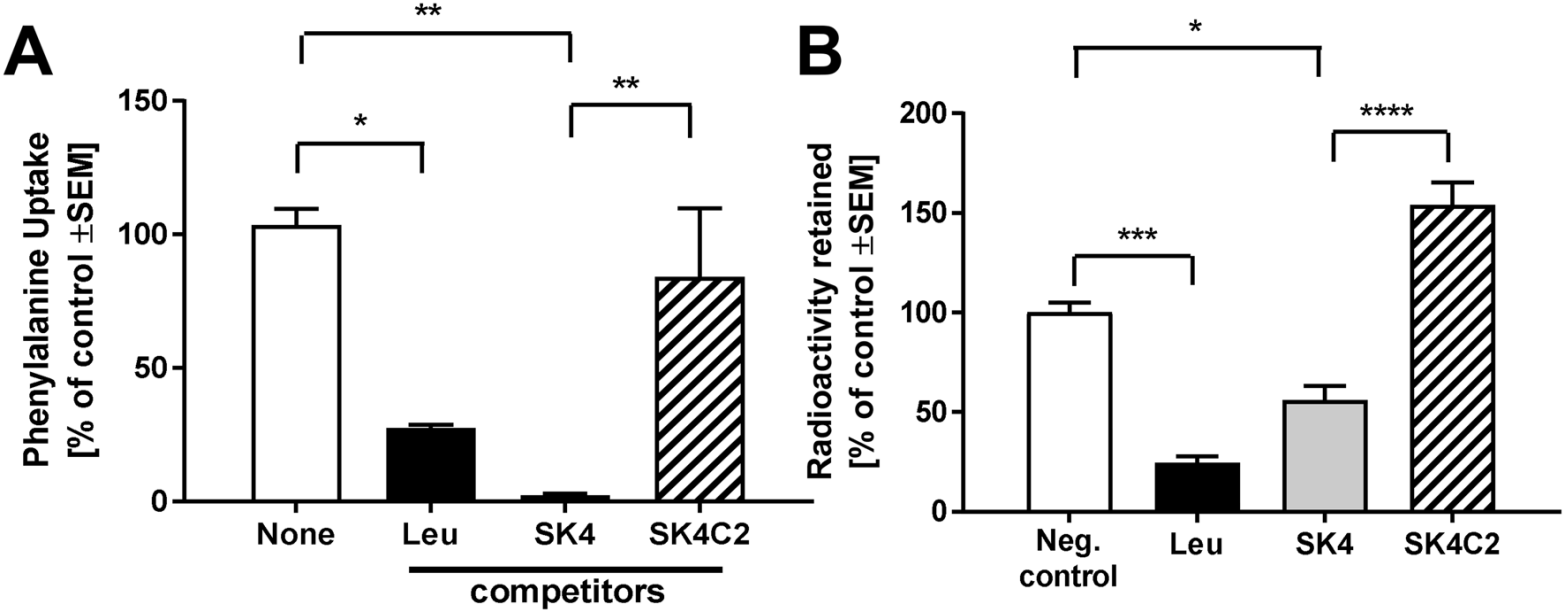
SK4 transport by LAT1. The transport of SK4 and SK4C2 by LAT1 was assessed in HEK293 cells transfected with human LAT1. Radiolabeled ^3^[H]-phenylalanine was used as established LAT1 substrate. **a** Cells were exposed to ^3^[H]-phenylalanine for 3 min in the presence or absence of competitors (1 mM). Then, the cells were washed, lysed and analyzed for their radioactive content. Uptake was normalized to the data obtained in the absence of competitors. **b** In a trans-stimulation assay, HEK293 LAT1 cells were preloaded with ^3^[H]-phenylalanine. Then, they were incubated in the presence of potential LAT1 substrates. The known substrate Leu was used as positive control of a compound that drives the antiporter transport cycle and, thus, accelerates the emptying of cells of LAT1 substrates such as phenylalanine. The cell emptying capacity via LAT1 was then compared for SK4 and SK4C2. Data are expressed as mean ± SD (*n* = 3) of three independent experiments performed in triplicate. Significantly different from indicated condition: *(*P* < 0.05), **(*P* < 0.01), ***(*P* < 0.001), ****(*P* < 0.0001)

To prove the validity of the “LAT1 substrate” design hypothesis, we assessed the properties of SK4 and the control compound. As expected for a LAT1 substrate, SK4 competed strongly with the phenylalanine uptake of cells overexpressing LAT1, and significantly increased the export of phenylalanine from preloaded cells in the trans-stimulation assay. SK4C2 (same iron chelator features as SK4, but lacking the amino acid group to be recognized by LAT1) was not transported at all (Fig. 4a, b).

### Direct iron-chelating properties of SK4

**Fig. 5:**
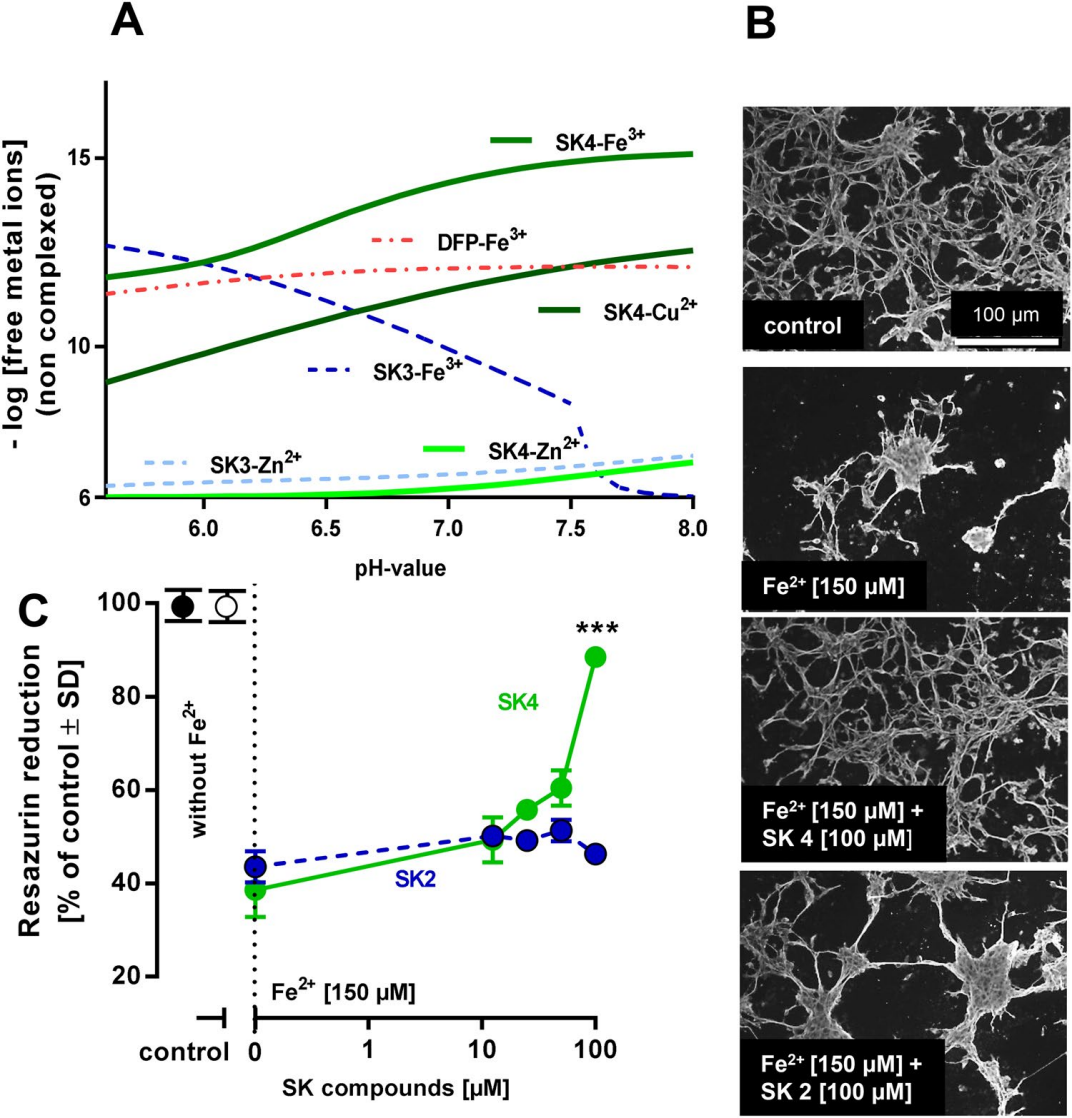
Iron chelating properties of SK4. **a** Chelation power of ligands for the studied metals (combinations specified in the graph) between pH 5.5 and 8. The *y*-axis shows the concentration of metal non-complexed to the ligand at the given conditions (− log[M^n+^]_nc_, [L]_tot_ = 0·M, ([M]_tot_  = 1·M), taking into account the experimentally determined protonation constants of the ligands, the stability constants of their metal complexes, and the hydrolysis constants of the metals. Thus, a value of 6 indicates no complexation by the given ligand (i.e., a free concentration of the metal ion of 1 µM), while a value of 12 indicates that 1 pM is non-complexed. Thus, the higher the value, the stronger the chelator. Note that the line for the SK2-Fe^3+^ complex is not shown in the diagram, as it runs continuously at 6 (no complex formation in the pH range shown). **b** Protection of neuronal cells from iron toxicity. Human dopaminergic neurons (LUHMES) were incubated with FeSO_4_ (150 µM) and SK2 or SK4 at the concentrations indicated for a period of 4 days. For visualization of cell morphology, cells were stained with an anti-β-III-tubulin antibody. Control cells showed a typical neuronal network with many fine neurite processes between the cells. Fe^2+^ led to cell loss and clumping of the remaining cells. This was prevented by SK4, but not SK2. The width of images corresponds to 200 μm in original cultures. **c** In experiments performed as in **b**, viability was assessed by the analysis of resazurin reduction. Data are means ± D of 3 independent experiments. There was a highly significant statistical difference at 100 µM (*P* < 0.001; ANOVA with Dunnett’s post hoc test)

SK4′s chelation properties towards Fe(III), Cu(II) and Zn(II) were determined by spectrophotometric titrations versus pH and compared to the reference standard DFP. As the basis for this, the protonation constants (pKa values) of SK4 were determined (Fig. S6), as chelation properties are dependent on the protonation state of the chelator, and as pKa values for various HOPO-related structures are known to show a broad range of variation (Fig. S7). The co-ordination models and stability constants of the formed complexes with metals (Fe(III), Cu(II) and Zn(II)) were then determined and the chelation potency/efficacy (displayed in terms of non-complexed metal remaining in solution (log[M^n+^]_nc_) was calculated under defined conditions (20-µM chelator, 1-µM metal ion) within the range of pH values relevant for cells (pH 5.5–pH 8) (Fig. 5a). SK4 showed stronger complex formation with Fe_3+_ than DFP (Fig. 5a), but exhibiting the same 3,4-HOPO complexing moiety, especially at higher pH values (as found e.g. within mitochondria). SK4 also showed affinity for Cu^2+^ ions, but almost none for Zn^2+^. Data were also obtained for compound SK3, based on the 1,2.HOPO scaffold, to exemplify the pH dependence of complex formation with Fe^3+^. The lower pKa values of this ligand favor its chelation properties at lower pH values.

Physicochemical studies in simple medium (0.1-M NaClO_4_ in this study) give critical information about the metal-chelating ability of ligands, but those cannot be extrapolated to complex biological systems (cells; brain) without caution. We, therefore, also tested the chelation capacity of SK2 and SK4 in a cellular model. Neuronal cultures were exposed to a FeSO_4_ solution, known to trigger cell death. SK4 protected neurons from iron-induced neurodegeneration. SK2 did not protect the neurons under similar conditions (Fig. 5b, c), in accordance with its lack of iron chelation capabilities under these experimental conditions. In this direct iron exposure test, the biological features (= cell protection) of SK4 and SK2 were obviously very different. Moreover, the radical scavenging property of SK2 was not of sufficient relevance to protect cells from externally added iron ions.

### Protection of neurons from MPP^+^ toxicity by SK compounds

**Fig. 6:**
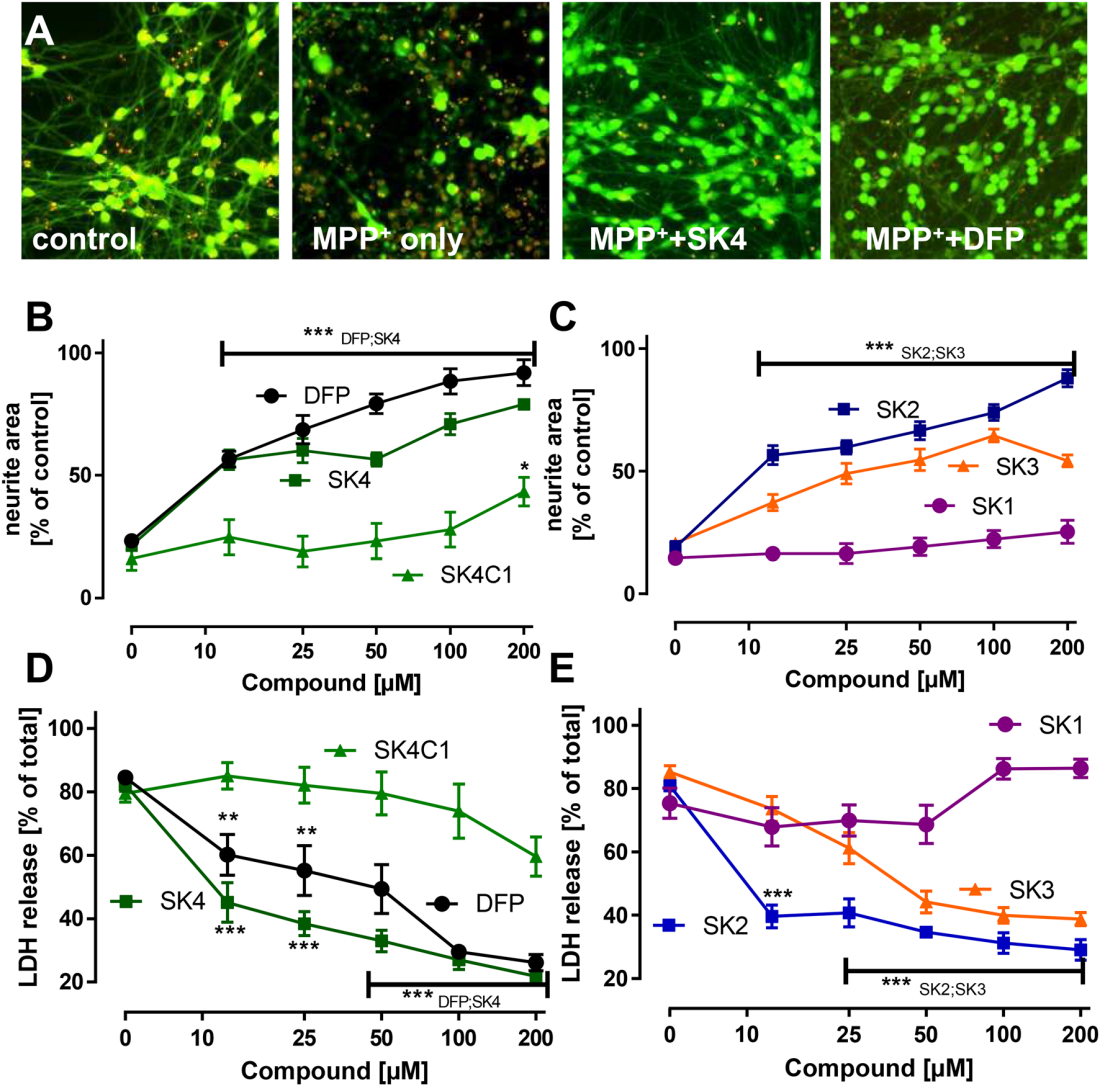
Protection of neurons from MPP^+^ toxicity by SK compounds. **a** Differentiated LUHMES neurons were treated on day 6 (d6) with MPP^+^ (5 μM) in the presence or absence of SK4 (200 μM) or DFP (200 μM). After 72 h, the cells were stained with calcein-AM (green stain of live cells) and H-33342 (red stain for live and dead cells). Representative images are shown. The width of one image corresponds to 300 μm of the original cultures. **b**/**c** Experiments were performed as described in **a**, but neurite integrity was quantified by automated microscopy as alternative endpoint **d**/**e** Experiments were performed as described in **a**–**c**, but the release of lactate dehydrogenase (LDH) was measured as death endpoint. Data are means + SEM of three independent experiments

We further characterized the compounds in the well-established parkinsonian MPTP/MPP^+^ model. As reported previously (Krug et al. 2014), dopaminergic neurons exposed to 5 μM of the mitochondrial toxicant MPP^+^ undergo apoptosis within 72 h, and this cell death can be prevented by iron chelators (Poltl et al. 2012). Cell death induced by MPP^+^ in our model system was assessed by the reduction of the neurite area and an increase in LDH release (Fig. 6a). SK2 and SK4 protected neurons at > 10 µM, and protection was similar to that of DFP. SK3 was less potent; whereas, SK1 completely lacked the ability to protect neurons from MPP^+^-induced cell death (Fig. 6b–e). SK4C1 also showed a very poor protecting activity (minor effect at ≥ 200 µM) (Fig. 6b, d). Furthermore, we found in an additional experiment that SK4 and DFP rescued dopaminergic neurons even when applied 2 h after the toxicant (Fig. S8).

Protection of neurons from 6-hydroxydopamine toxicity by SK4.

**Fig. 7:**
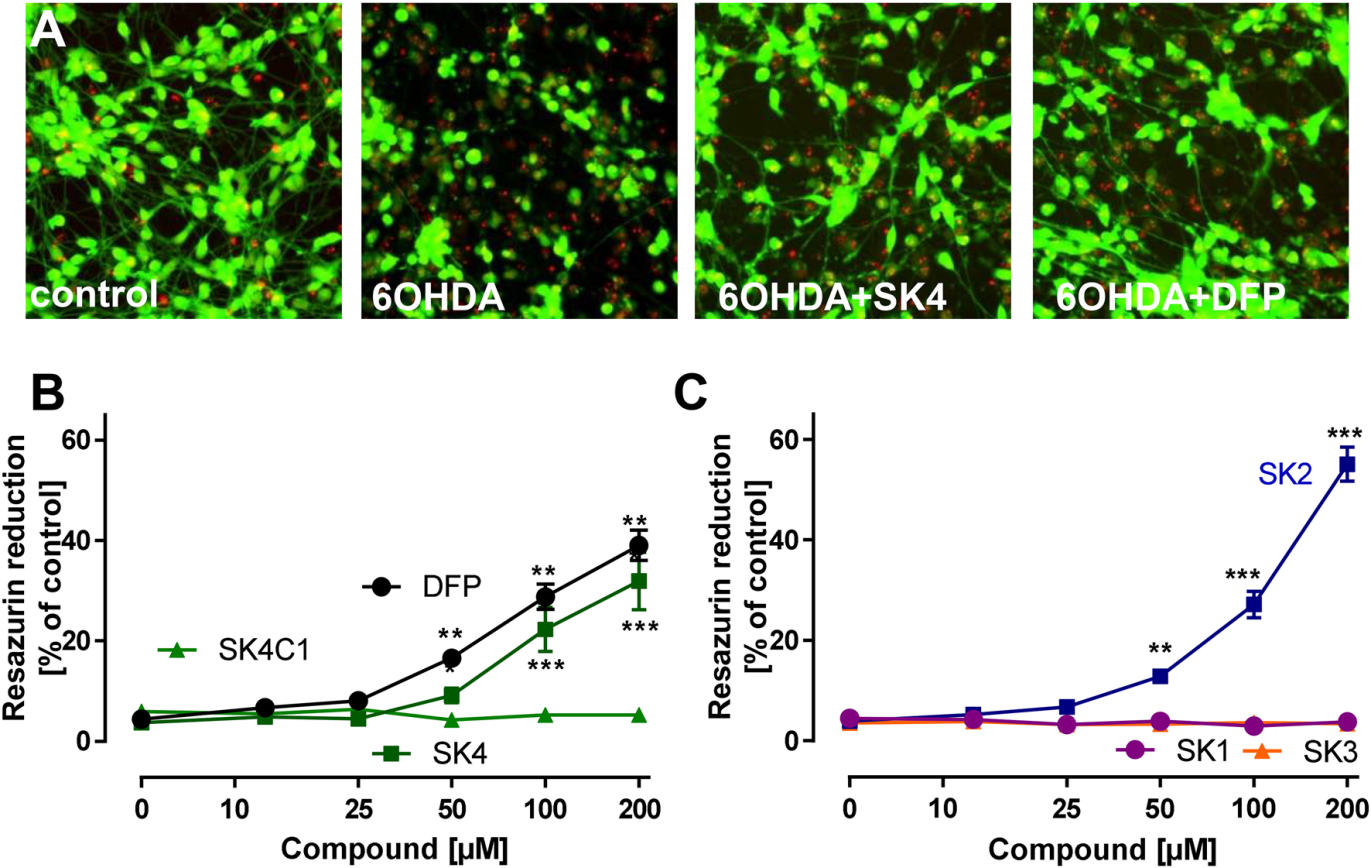
Protection of neurons from 6-hydroxydopamine toxicity by SK compounds. **a** Differentiated LUHMES neurons were treated on day 6 (d6) with 6-OHDA (100 μM) in the presence or absence of SK4 (200 μM) or deferiprone (DFP; 200 μM). After 18 h, the cells were stained with calcein-AM (green stain of live cells) and H-33342 (red stain for live and dead cells). Representative images are shown. The width of one image corresponds 300 μm of the original cultures **b**/**c** Neuroprotection against 6-OHDA-induced neuronal damage of LUHMES (d6) cells was tested as in **a**, but resazurin reduction was used as quantitative endpoint. Data are means + SEM of three independent experiments

A further well-established model is based on the incubation of dopaminergic neurons with 6-hydroxydopamine (6-OHDA). The corresponding in vivo model shows several key characteristics of neurodegeneration in PD (Lindholm et al. 2007; Salari and Bagheri 2019; Ungerstedt 1973). The damage initiated by 6-OHDA involves oxidative stress to neurons, with endogenous iron levels being important contributors to this damage mechanism (Shachar et al. 2004; Workman et al. 2015; Youdim et al. 2004). When neurons were incubated with 6-OHDA, cells lost their neurites within 24 h; while, somata stayed intact and viable (Fig. 7a). This specific neurite degeneration was significantly reduced by SK4 (≥ 100 µM) and DFP (≥ 50 µM) (Fig. 7a, b). Also SK2 reduced the damage (at ≥ 50 µM); at very high concentrations (200 µM), its effect was more pronounced than the profile produced by either SK4 or DFP (Fig. 7c). All other compounds tested did not show any effect on neurite loss following 6-OHDA exposure (Fig. 7b, c).

### Protection of neuronal organoids by SK4 from ferroptosis

**Fig. 8:**
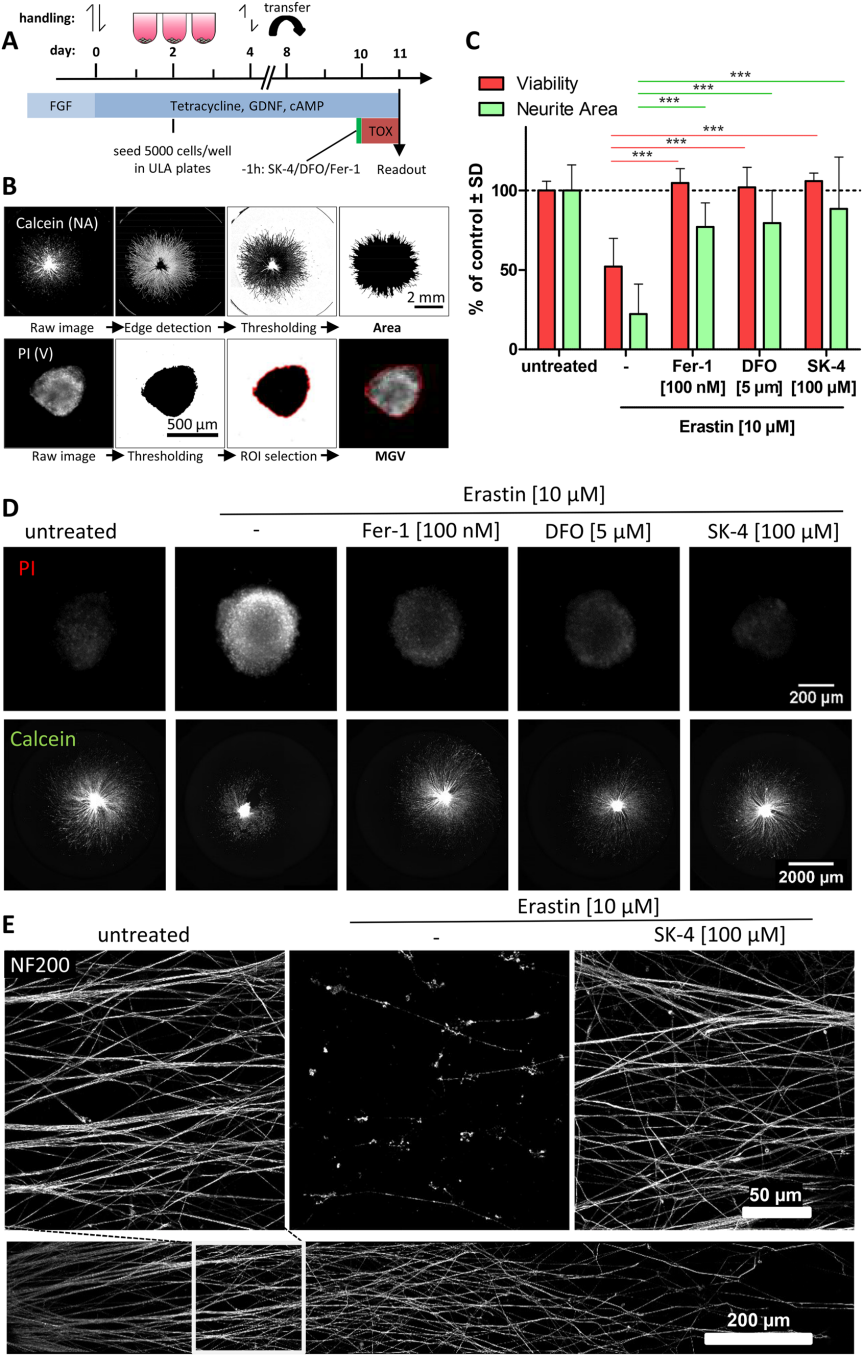
Protection by SK4 from erastin-induced ferroptosis in neuronal organoids. **a** Generation of LUHMES organoids: LUHMES (pre-differentiated for 2 days) were left to self-aggregate (d2–d8), and the resultant spheres were then plated. After 48 h of neurite outgrowth from the organoids (d8–d10) they were treated with erastin (10 μM) for 24 h. Optionally, they were preincubated for 1 h with ferrostatin-1, or the iron chelators desferoxamine (DFO), or SK4. Finally, organoids were stained with calcein-AM and propidium iodide to assess survival endpoints. **b** Workflow (and exemplary primary and processed images) to quantify the total neurite area (calcein staining) and the cell viability (propidium iodide (PI) fluorescence). **c** The plated organoids were treated for 24 h with either erastin alone (10 μM) or together with ferrostatin-1 (100 nM), desferoxamine (5 μM) or SK4 (100 μM). Data are means ± SD (three independent experiments with at least three technical replicates each). Statistical significance was determined by one-way ANOVA with Dunnet‘s post hoc test in comparison to erastin treatment. ***P < 0.001. **d** Representative images of neurite areas stained with PI and calcein under various conditions. **e** Organoids were fixed and immunostained for neurofilament heavy chains (NF200). Images were recorded by confocal microscopy. Representative images are shown for cultures exposed to erastin in the presence or absence of SK4

To gain insight into the potential protective effect of SK4 on the tissue level, we established a 3D neuronal model for erastin-triggered neurodegeneration. Neuronal spheroids were generated within 10 days. After complete differentiation and neurosphere formation, the organoids were pretreated with protective compounds for 1 h and then exposed to erastin for 24 h (Fig. 8a). To analyze the damage, viable structures were visualized by staining with calcein-AM or immunostaining of neurofilament heavy chains (NF200). The overall neurite area was analyzed to assess putative protective effects by the SK compounds (Fig. 8b–d). The anti-ferroptotic agent Fer-1 (Miotto et al. 2020) protected spheroids from neurodegeneration. A similar protective effect was also observed for the iron chelators DFO and SK4 (Fig. 80c, d).

## Discussion

Here, we present data on the physicochemical characterization as well as cellular investigations of novel iron chelators produced in our laboratories. Neuroprotective properties were investigated using established PD- and iron overload in vitro models that utilized human LUHMES neuronal cultures. The cells were challenged with various toxicants that recapitulate aspects of PD, including dopaminergic cell death, oxidative stress and mitochondrial functional defects (Schildknecht et al. 2017). Potential iron-chelator drug candidates were also protective in a neuronal ferroptosis model, as well as in a direct iron overload paradigm. As SK4 consistently provided neuroprotection against various cytotoxic conditions, our work suggests that this chemical is a useful new tool to further elucidate the mechanistic role of iron in neurodegenerative processes. Moreover, it indicates new strategies to develop brain-targeted metal chelators.

Iron chelators have been deemed therapeutically useful in PD and other neurodegenerative conditions (Dunaief 2011; Joppe et al. 2019; Kaindlstorfer et al. 2018; Ndayisaba et al. 2019). However, their exact effects on brain pathophysiology have not been fully elucidated. One important hypothesis is that the abnormally high labile iron levels in the nigrostriatal system of PD patients contribute to a heightened pro-oxidant environment that impacts on vulnerable dopaminergic neurons found in the SNpc. In this regard, iron-dependent oxidative stress has been demonstrated in vitro, in parkinsonian animal models and also in post-mortem brains of PD patients (Devos et al. 2014; Kaur et al. 2003; Ward et al. 2014; Zheng et al. 2005). In this context, it is important to note that iron-dependent pathology may be triggered without additional iron to an experimental system or human tissue. Brain cells contain mM concentrations of overall iron, mostly bound to ferritin (Reinert et al. 2019; Workman et al. 2015). If only a small fraction of such iron is mobilized, i.e., transferred to the redox-active labile iron pool (LIP), iron-dependent toxicity, and an iron-dependent form of programmed death termed ferroptosis, can ensue (Stockwell et al. 2017). Alternatively, iron can change its role in a cell without increasing ferric iron concentrations. When the overall oxidant–antioxidant balance is shifted, normally innocuous iron concentrations can take on pathological roles, where this can be counteracted again either by activating the cell’s antioxidant defence system (Liddell and White 2018) or by the use of iron chelators. This background explains why iron plays a role in several toxin-based models of mitochondrial impairment and oxidative stress (Poltl et al. 2012; Santiago et al. 1997). In line with this, SK4 fully protected differentiated LUHMES cells against the differently acting toxicants MPP^+^ and 6-OHDA.

Although it remains unresolved, whether oxidative stress can be considered a primary cause of the neuronal death seen in neurodegenerative disease patients or as a bystander in the progressive neurodegenerative cascade (Andersen 2004), increased iron levels have been shown to directly catalyze ROS production during dopamine synthesis (Rhodes and Ritz 2008). Many forms of neurodegenerations are also accompanied by the production of peroxynitrite (Schildknecht et al. 2013). This highly reactive chemical species, formed by recombining NO with superoxide, interacts directly with a variety of biological targets to exert irreversible damage to all classes of biomolecules (Weidinger and Kozlov 2015). As the cellular damage is strongly driven by peroxynitrite in some model systems, it is important to verify, whether a novel agent can be regarded as a direct scavenger of this molecule or other reactive oxygen/nitrogen species. However, such issues for complicating interpretation of compounds’ mechanism of action do not apply to LUHMES culture systems. This is since these cells do not produce endogenous peroxynitrite due to their very low NO synthase activity (Schildknecht et al. 2015). Thus, our data showing that SK4 is not a potent antioxidant is a clear indication that this compound’s ability to protect against MPP^+^ and 6-OHDA neurotoxicity must be due to its iron chelation effects.

In the area of experimental pharmacology and for proof-of-concept studies, it is rather important that iron chelators have no other targets and activities. This is the only way to provide clear evidence for a causal role of iron in neurodegeneration models. In this context, the finding that SK4 does not interact with all major reactive oxygen species is of importance. Moreover, the protection from cell death in multiple tests by the non-antioxidant iron chelator SK4 strongly suggests a causal involvement of iron in all these models of neurodegeneration. For a novel metal chelator to be regarded as having potential clinical value for treating the brain degenerating disorder PD, the drug should inherently possess the ability to effectively cross the BBB. Several FDA-approved iron chelators, including deferoxamine (Okauchi et al. 2009) and DFP (Boddaert et al. 2007) may cross the BBB to some extent. Drugs that accumulate passively in brain tissue or that are actively transported across the BBB are of interest, as long-term iron chelation may have adverse side effects in the body periphery. SK1-4 were designed to be substrates of the LAT1 transporter, which is highly expressed in brain capillary endothelial cells. This carrier is responsible for transporting large neutral amino acids, including phenylalanine, tyrosine and leucine, across the BBB in a sodium-ion-independent manner (Matsuo et al. 2000). The same transport system allows transfer of the hydrophilic drug levodopa, a precursor for dopamine, into the brain (Smith and Takasato 1986), with our compounds that were designed to mimic the chemical features of L-DOPA, to be recognized by LAT1. We experimentally validated our structural design hypothesis by showing that SK4 is transported into LAT1 expressing cells. In this respect, the SK compound differs from the clinically approved iron chelators, desferrioxamine (DFO), DFP, and deferasirox (ICL-670), which rather transgress the BBB via passive diffusion (Zhou et al. 2012).

Iron chelation therapy has its origins in the treatment of iron-overload syndromes such as the thalassemias. For these inherited blood disorders, the reduction of an iron overload has clearly proven to be clinically beneficial (Mobarra et al. 2016). However, the scenario is less clear for PD, where clinical trials have only been partially successful. One explanation may be that current drug delivery methods are not optimized for yielding high chelator concentrations in the CNS. More targeted drug delivery methods hold clear advantages for improving clinical outcomes, including reducing drugs’ side effects in the periphery. Our study provides proof-of-concept for a novel delivery approach. Other drug properties may also play important roles. Each metal chelator has a specific “activity” fingerprint, concerning the affinity to ferric iron vs ferrous iron vs several other metal ions (Cu^+^, Zn^2+^, etc.), concerning the Ph dependence of such affinities, and the competition with cellular iron-chelating structures, especially in the active center of metalloenzymes. In this regard, it may prove to be a neuroprotective advantage to inactivate certain prolyl hydroxylases by removing their iron (Karuppagounder et al. 2016); on the other hand, it may be detrimental to inactivate catalase or mitochondrial respiratory chain complexes. For this reason, drug candidates appearing similar in terms of their interaction with ferric iron may prove to have largely different drug properties. In the absence of more information on what the optimal profile should look like, it is important to have a large panel of diverse iron chelators available for pharmacological studies. The novel SK compounds, and in particular SK4, contribute to this important objective.

## Electronic supplementary material

Below is the link to the electronic supplementary material.

Supplementary file1 (PDF 1097 kb)

## Abbreviations

ALP: Autophagy–lysosomal pathway
AD: Alzheimer’s disease
α-SYN: Alpha-synuclein
BBB: Blood–brain barrier
BSA: Bovine serum albumin
CSF: Cerebrospinal fluid
DFO: Desferoxamine
DFP: Deferiprone
DHE: Dihydroethidium
DHR: Dihydrorhodamine
DM: Differentiation media
FBS: Fetal bovine serum
FGF-2: Fibroblast Growth Factor 2
GDNF: Glial-derived neurotrophic factor
HOPO: Hydroxypyridinone
LAT1: Large neutral amino acid transporter
LDH: Lactate dehydrogenase
Mp: Melting point
MPP^+^: 1-Methyl-4-phenylpyridinium
MPTP: Methyl-4-phenyl-1, 2, 3, 6-tetrahydropyridine
PD: Parkinson’s disease
PFA: Paraformaldehyde
PLO: Poly-l-ornithine
PM: Proliferation media
PKAN: Pantothenate kinase-associated neurodegeneration
UDPRS: Unified Parkinson Disease Rating Scale
UV: Ultraviolet
ROS: Reactive oxygen species
RT: Room temperature
SNpc: Substantia Nigra pars compacta
6-OHDA: 6-Hydroxydopamine
