# Supplementary material for: Design and evaluation of bi-functional iron chelators for protection of dopaminergic neurons from toxicants
Source: Arch Toxicol. 2020 Jun 30;94(9):3105–23. doi: 10.1007/s00204-020-02826-y (PMC7415766; doi:10.1007/s00204-020-02826-y)
Supplement: Supplementary file 1 — Supplementary file1 (PDF 1097 kb) [file 204_2020_2826_MOESM1_ESM.pdf]

Supplementary information (SI)

Neuroprotection by novel hydroxypyridinone-based metal chelators in various cellular models of parkinsonian neurodegeneration

Simon Gutbier[1], Sotiris Kyriakou[2*], Stefan Schildknecht[1], Anna-Katharina Ückert[1], Markus Brüll[1], Frank Lewis[2], David Dickens[4], Liam Pearson[4], Joanna L. Elson[5], Sylvia Michel[3], Véronique Hubscher-Bruder[3], Jeremy Brandel[3], David Tetard[2#], Marcel Leist[1#] and Ilse S. Pienaar[6#]*

Table of Contents

Supplementary methods (with supplementary figures 1-5)

- Design rationale
- Outline of synthesis strategy

Supplementary Fig. 6

Supplementary Fig. 7

Supplementary references

Supplementary methods

Design rationale

Decrease of brain iron levels is considered a promising approach to treat neurodegenerative diseases such as PD. Currently the concept is assessed in several clinical trails using DFP, as it is known to cross the BBB (Fredenburg, Sethi, Allen & Yokel, 1996). However, an increased ratio of central nervous system (CNS) to peripheral drug concentration would be desirable. Several approaches to increase delivery to the central nervous system, i.e. hijacking known transport systems by coupling drugs to glucose or transferrin, are described (Roy, Preston, Hider & Ma, 2010; Scott et al., 2011). A transporter that is known to improve drug penetration over the BBB is LAT1 (Zheng, Youdim, Weiner & Fridkin, 2005). Exploitation of LAT1 as a transporter to help chelators reach the brain has been studied on one derivative of clioquinol called M10 (Zheng, Youdim, Weiner & Fridkin, 2005). This compound is in essence a derivative of L-α-amino acids that are natural substrates of LAT1. However, the side-chain of M10 is significantly different from that of the natural substrates of LAT1. It may or may not therefore suffer from poor brain uptake. To the best of our knowledge, the BBB penetration of M10 has never been reported. L-DOPA is known to be a substrate of LAT1. L-DOPA contains a catechol group that is isoelectronic with HOPOs and of similar size. We therefore hypothesized that creating derivatives of L-DOPA where the catechol group is replaced by a metal chelator moieties of similar size and electron distribution inspired by DFP would create compounds likely to be LAT1 substrates and therefore capable of reaching the CNS while maintaining the neuro-protective property of DFP.

Additionally, it is known that L-DOPA is converted by the catechol O-methyl transferase (COMT) enzyme into 3-OMD (Kiss & Soares-da-Silva, 2014). Any chelator used as treatment of PD must not have its coordinating atoms methylated by COMT and thus lose potency. 3-hydroxy-4(1H)-pyridinones are known to be extremely poor substrates of COMT, thus making them attractive candidates largely unaffected by that enzyme in the CNS (Singh et al., 1992). However, 3-hydroxy-4(1H)-pyridinones are known to be glycosylated on their hydroxyl group by metabolic processes (Singh et al., 1992), leading to loss of chelation ability. It is therefore useful to also study other hydroxypyridinone isomers as their metabolic conversions may be less detrimental. The compounds tested are labelled SK1-SK5 and are depicted in Figure 1.

Outline of synthesis strategy

Chelators SK1, SK2, SK3, SK5 were synthesized as racemic mixtures. The previously known compounds (SK2 and SK5) were synthesized using modified literature methods (Harris, 1976; Harris & Teitei, 1977). Novel racemic compounds SK1 and SK3 were synthesized by adapting literature methods. SK4 (absolute configuration S) was synthesized as depicted in Fig. S1 from maltol and Boc-protected asparagine (Harris, 1976; Kawasuji et al., 2013; Radić Stojković, Piotrowski, Schmuck & Piantanida, 2015) and described in detail in the material and methods part.

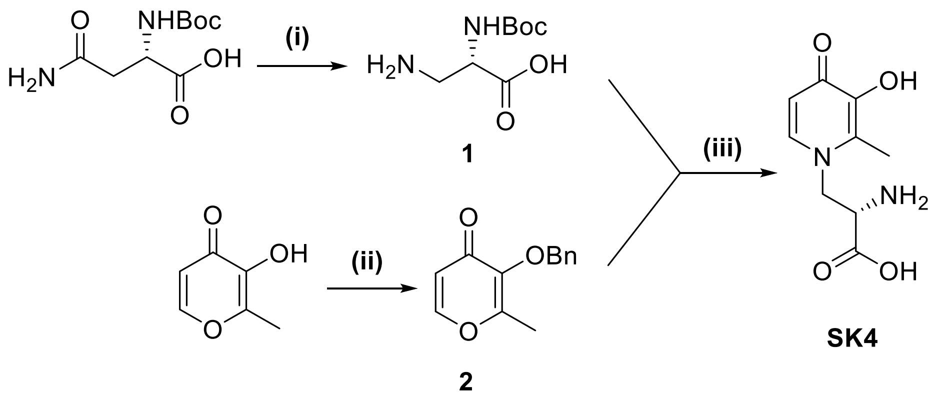

Fig. S1: **Synthesis of SK4.** Reagents and conditions: (i) a) N-Boc-L-Asn, Iodosobenzene diacetate, EtOAc: MeCN: H_2O, RT, 4hr, 76%; (ii) BnBr, K_2CO_3, DMF, 80°C, 1 h, 76%; (iii) a) EtOH: H_2O, 8 days, b) conc. HBr, reflux, 20 min, c) NH_3, pH 5, 5°C, 72 h, 63%.

Synthesis of precursor P1:

In a solution mixture composed of ethyl acetate (24 mL), acetonitrile (24 mL) and water (12 mL), *N*-Boc-*L*-asparagine (5.0 g, 21.5 mmol) and iodosobenzene diacetate (8.32 g, 25.8 mmol) were added. The resulting slurry, was stirred at 16°C for 30 min and then at 20°C for 4 h. Upon completion of the reaction, the mixture was cooled at 0°C for 15 min forming a white salt which was collected by filtration. The filter-cake was then washed with cooled ethyl acetate (30 mL) affording compound 1 as a white solid (3.34 g, 16.35 mmol, 76%). Mp: 203-207°C [lit: 207-212°C]. ^{1}H-NMR (400 MHz, DMSO-d_6) δ_H= 1.38 (s, 9H), 2.67-2.73 (m, 1H), 2.99-3.02 (m, 1H), 3.57-3.61 (m, 2H), 6.16 (s, br, 1H); ^{13}C-NMR (100 MHz, DMSO-d_6) δ_C= 28.6, 41.2, 51.3, 78.7, 155.6, 171.6.

Synthesis of precursor P2:

In a solution of maltol (10 g, 79.26 mmol) in *N, N*- dimethyl formamide (100 mL), benzyl bromide (9.42 mL, 79.26 mmol) was added and the solution mixture was stirred at 80°C for 15 min. Then, a sample of potassium carbonate (12.05 g, 87.18 mmol) was added to the reaction mixture and the final mixture was heated at 80°C for a further 1 h. Upon completion of the reaction, the excess of inorganic salt was removed by filtration and the filtrates were concentrated under reduced pressure. The resulting residue was dissolved in tetrahydrofuran (50 mL) and any remaining of the inorganic salt was removed by filtration. Then, the filtrates were concentrated under reduced pressure affording the titled compound as a viscous orange oil (16.28 g, 75.29 mmol, 95%). ^{1}H-NMR (400 MHz, $CDCl_3$) δ_H= 2.07 (s, 3H), 5.13 (s, 2H), 6.37 (d, J= 5.6 Hz, 1H), 7.28-7.39 (m, 5H), 7.60 (d, J= 5.6 Hz, 1H); ^{13}C-NMR (100 MHz, $CDCl_3$) δ_C= 14.6, 73.3, 116.8, 128.2, 128.3, 128.8, 136.7, 143.6, 153.9, 159.8, 175.02.

Synthesis of SK1 and SK2:

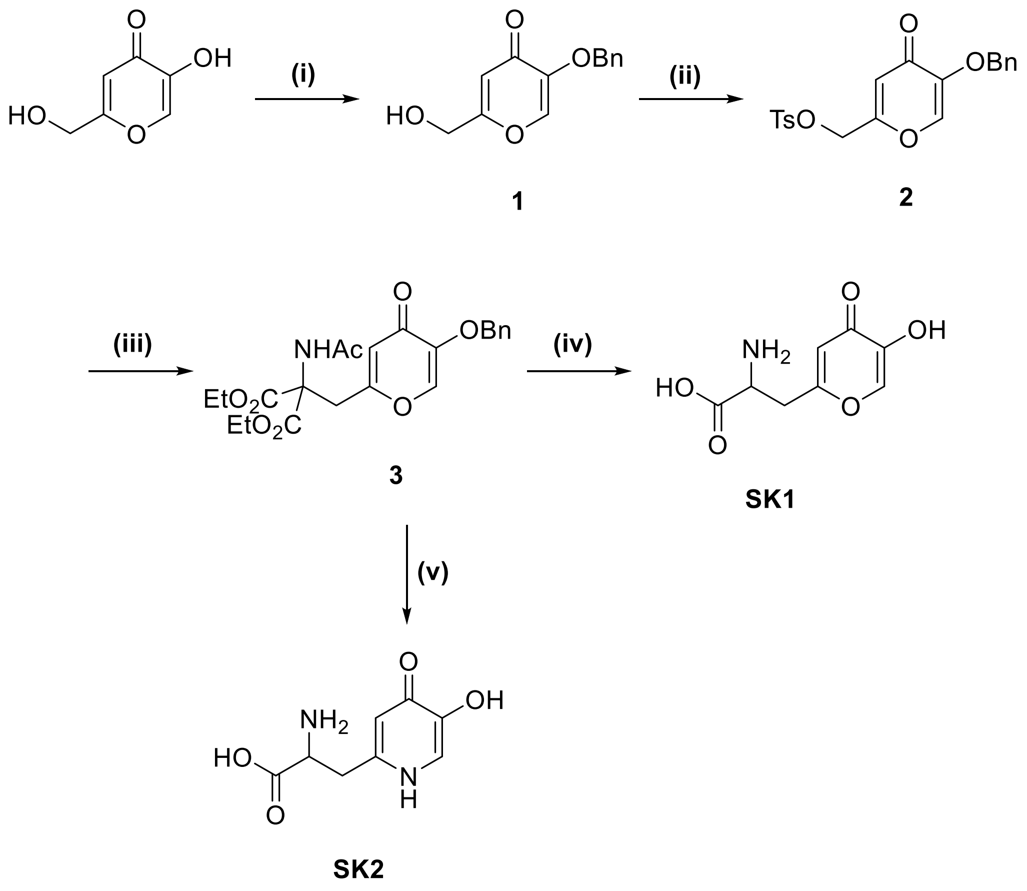

Fig. S2: Synthesis of SK1 and SK2. Reagents and conditions: (**i**) **a**) $NaOH_{(aq)}$, MeOH, 40 min, 110°C, **b**) BnBr, overnight, 120°C, 87%; (**ii**) TsCl, $NaOH_{(aq)}$, acetone, 20 min, RT, 94%; (**iii**) Diethyl acetamidomalonate, NaH (60% in mineral oil) $DMF_{(dry)}$, overnight, RT, 95%; (**iv**) **a**) conc. HCl, 180°C, 3 h, **b**) conc. NH_4OH, pH 5, 5°C, 89%; (**v**) **a**) conc. NH_4OH, 130°C, 3 h, **b**) conc. HCl, 180°C, 3 h, **c**) conc. NH_4OH, pH 5, 5°C, 91%.

SK1 and SK2 were synthesized starting from kojic acid in four steps of which the last reaction differed between SK1 and SK2 (Fig. S2).

5-benzyloxy-2-(hydroxymethyl)-4H-pyran-4-one (1) [1]

A sample of kojic acid (20 g, 141 mmol) was dissolved in methanol (80 mL) and mixed with a solution of sodium hydroxide (6.2 g, 155 mmol) in water (30 mL). The mixture was reflux for 40 min before the dropwise addition of benzyl bromide (19 mL, 155 mmol). The mixture was allowed to reflux overnight. Upon completion of the reaction, the solvents were removed under reduced pressure and the residue was taken up in dichloromethane (200 mL) and washed with aqueous solution of sodium hydroxide (5%, 2 x 100 mL). The organic extracts were then washed with water, brine, dried over magnesium sulphate and concentrated under reduced pressure to give the crude product as yellowish crystals. The crude product was recrystallized from isopropanol, dried overnight at 65°C affording the pure product as white crystals (29 g, 125 mmol, 87%). Mp: 126-129°C [lit: 128-130°C]. ^{1}H-NMR (400 MHz, DMSO-d_6): δH= 4.24 (d, J= 5.6 Hz, 2H), 4.89 (s, 2H), 5.70 (t, J= 5.6, 1H) 6.29 (s, 1H), 7.26-7.38 (m, 5H),

8.13 (s, 1H); ^{13}C-NMR (100 MHz, DMSO-d_6): δC= 65.5, 71.0, 111.6, 128.6, 128.7, 128.9, 136.6, 141.6, 147.1, 168.7, 173.8.

[5-benzyloxy-4-oxo-4H-pyran-2-yl]methyl 4-methylbenzene-1-sulfonate (2) [2]

A sample of 5-benzyloxy-2-(hydroxymethyl)-4H-pyran-4-one (1) (25 g, 108 mmol) was dissolved in acetone (350 mL) and stirred vigorously before tosyl chloride (21 g, 110 mmol) was added at RT. Then, a solution of sodium hydroxide (4.3 g, 108 mmol) in water (18 mL) was added and the resulting mixture stirred at RT for 20 min. The crude product was precipitated upon addition of water (150 mL) and was purified by recrystallization from methanol/water affording the pure compound as pale-yellow crystals (38 g, 98 mmol, 94%). Mp: 111-114°C [lit: 112°C]. ^{1}H-NMR (400 MHz, DMSO-d_6): δH= 2.47 (s, 3H), 4.77(s, 2H), 5.02 (s, 2H), 6.33 (s, 1H), 7.32-7.36 (m, 7H), 7.46 (s, 1H), 7.77 (d, J = 8 Hz, 2H) ppm; ^{13}C-NMR (100 MHz, DMSO-d_6): δC= 21.8, 66.0, 71.9, 115.5, 127.8, 128.1, 128.6, 128.9, 130.2, 132.3, 135.5, 141.5, 145.8, 147.4, 158.7, 174.0. HRMS (ESI) for $C_{20}H_{18}O_6S$: theoretical [M+H]: 387.0824, measured [M+H]: 387.0890.

1,3-diethyl 2-([5-benzyloxy-4-oxo-4H-pyran-2-yl]methyl)-2-acetamidopropane dioate (3)

In a solution of diethyl acetamidomalonate (10 g, 46 mmol) in dry N, N-dimethyl formamide (70 mL), under nitrogen atmosphere, sodium hydride (60% in mineral oil, 2 g, 83 mmol) was added in portions. Upon the evolution of hydrogen gas was ceasing, a sample of [5-benzyloxy-4-oxo-4H-pyran-2-yl]methyl 4-methylbenzene-1-sulfonate (2) (8.5 g, 22 mmol) was added to the solution mixture which was stirred overnight at room temperature and protected from moisture. Upon completion of the reaction, the solvents were removed under reduced pressure forming a brown slurry which was mixed with water (100 mL) and stirred vigorously. The resulting crude product precipitated as brown solid, collected by filtration, left to dry overnight and purified by recrystallization (acetone/petroleum ether 60:80) affording the pure compound as light orange crystals (9.21 g, 21 mmol, 95%). Mp: 117-120°C [lit: 117-118°C]. ^{1}H-NMR (400 MHz, DMSO-d_6): δH = 1.18 (t, J= 7.2 Hz, 6H), 1.93 (s, 3H), 3.41 (s, 2H), 4.14 (m, 4H), 4.90 (s, 2H), 6.10 (s, 1H), 7.37-7.44 (m, 5H), 8.15 (s, 1H), 8.51 (s, 1H); ^{13}C-NMR (100 MHz, DMSO-d_6): δC= 14.3, 22.4, 31.2, 62.8, 65.6, 71.0, 116.1, 128.7, 128.8, 129.0, 136.5, 142.0, 147.2, 163.2, 167.0, 170.4, 173.4. HRMS (ESI) for $C_{22}H_{25}NO_8$: theoretical [M+H]: 432.1655, measured [M+H]: 432.1656.

2-amino-3-(5-hydroxy-4-oxo-4H-pyran-2-yl)propanoic acid (SK-1)

A solution of concentrated hydrochloric acid (HCl) (40 mL) and 1, 3-diethyl 2-([5-benzyloxy-4-oxo-4H-pyran-2-yl] methyl)-2-acetamidopropanedioate (3) (5.1 g, 11.84 mmol) was heated at 180°C for 3 h. Upon completion of the reaction, the solvents were removed under reduced pressure forming a brown solid which was dissolved in water (20 mL). The solution was treated with charcoal, filtered and the pH of the filtrate was adjusted to 5.0 by the dropwise addition of concentrated ammonium hydroxide. The resulting solution was kept overnight at 5°C. White crystals were precipitated, collected, washed with water, acetone, petrol ether (60:80) and dried in the air affording the pure compound as white crystals (2.10 g, 10.54 mmol, 89%). Mp: 116-117°C. ^{1}H-NMR (400 MHz, D_2O/CF_3COOD 8:2): δH = 2.63-2.75 (m, 2H), 3.84 (t, J= 6.8 Hz, 1H), 5.97 (s, 1H), 7.45 (1H, s); ^{13}C-NMR (100 MHz, D_2O/CF_3COOD 8:2): δC= 32.9, 50.0, 116.4, 119.3, 142.5, 144.2, 169.4, 175.5. HRMS (ESI) for $C_8H_9NO_5$: theoretical [M+H]: 199.0713, measured [M+H]:199.0710.

2-amino-3-(5-hydroxy-4-oxo-1,4-dihydropyridin-2-yl)propanoic acid (SK-2) [1]

A portion of 1,3-diethyl 2-([5-benzyloxy-4-oxo-4H-pyran-2-yl]methyl)-2 acetamidopropane dioate (3) (4.2 g, 9.73 mmol) was mixed with a solution of concentrated ammonium hydroxide (25 mL) and the mixture was heated for 5 h in a stainless-steel pressure vessel at 120°C. Upon completion of the reaction, the mixture was evaporated to dryness and the resulting solid was dissolved in a solution of concentrated HCl (30 mL). The resulting mixture was heated at 180°C for 3 h. The solvents were evaporated and the resulting crystals were dissolved in water (20 mL). The solution was treated with charcoal, filtered and the pH was adjusted to 5.0, using ammonia solution. The resulting solution was kept overnight at 5°C forming white crystals which were collected, washed with water, acetone, and light petroleum and dried affording the pure compound as white crystals (1.76 g, 8.9 mmol, 91%) Mp: 230-234°C [lit: >250°C]. ^{1}H-NMR (400 MHz, D_2O/CF_3COOD 8:2): δH= 2.69-2.83 (m, 2H), 3.73 (t, J= 6.8 Hz, 1H), 6.53 (s, 1H), 7.3 (s, 1H); ^{13}C-NMR (100 MHz, D_2O/CF_3COOD): δC= 30.1, 51.2, 113.6, 116.4, 142.5, 143.4, 168.7, 169.0. HRMS (ESI) for $C_8H_{10}N_2O_4$: theoretical [M+H]: 198.0640, measured [M+H]: 198.0870.

Synthesis of SK3:

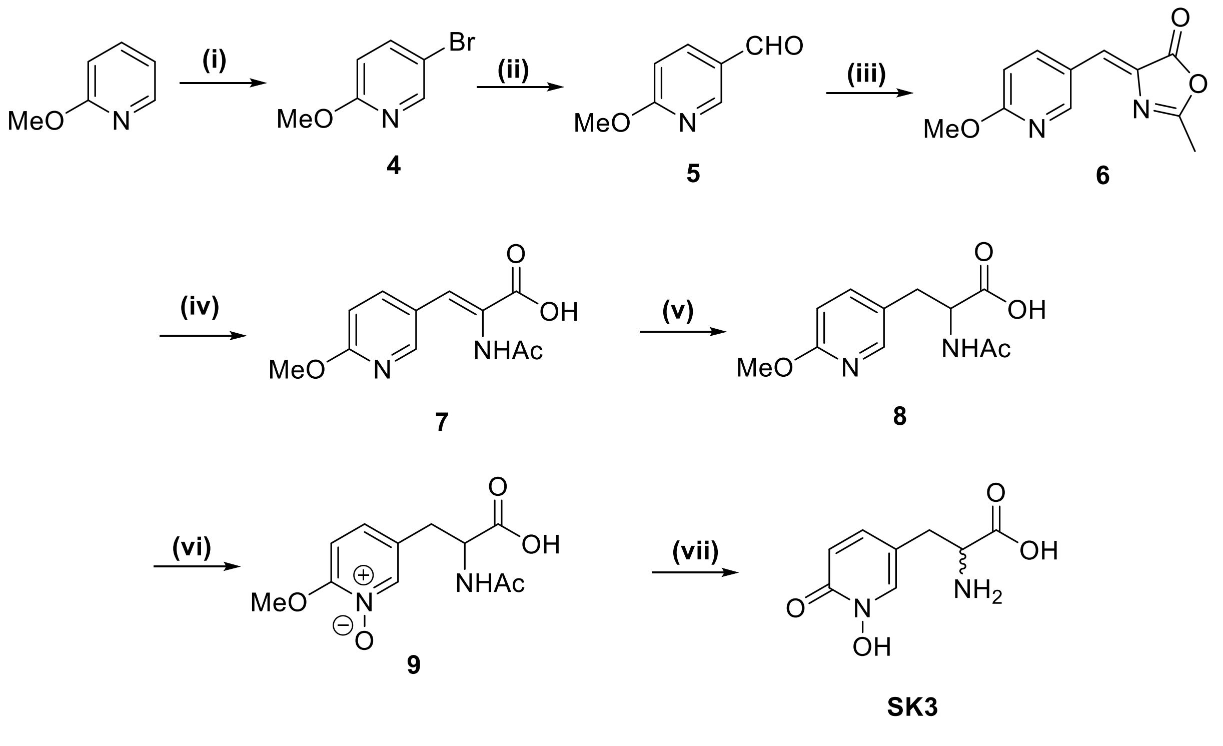

Fig. S3: Synthesis of SK3: Reagents and conditions: (**i**) N-Bromosuccinimide, CH_3CN, 90°C, 2 h, 18%; (**ii**) *n*-BuLi, Et_2O, DMF, -35°C, 7 h, 67%; (**iii**) *N*-acetyl glycine, AcONa, Ac_2O, 130°C, 4 h, 63%; (**iv**) H_2O, reflux, 4 h, 81%; (**v**) H_2,10% Pd/ C (*cat*), MeOH, RT, 9 h, 53%; (**vi**) *m*-CPBA, DCM, MeOH, RT, 48 h, 77%; (**vii**) conc. HCl, reflux, 1 h, 54%.

5-bromo-2-methoxypyridine (4) [3]

In a suspension of 2-methoxypyridine (15 g, 138 mmol) in acetonitrile (415 mL), N-bromosuccinimide (30 g, 169 mmol) was added and the resulting mixture was refluxed for 20 h. Upon completion of the reaction, as it was indicated by TLC (SiO_2, eluent: Petroleum Ether 60-80: Ethyl acetate, 8:2), the mixture was filtered over a pad of silica. The solvents were evaporated, under reduced pressure, affording the crude product as orange oil which was then purified by an automated flash chromatography column (Biotage IsoleraOne^{TM}); Rf= 0.83 (Petrol Ether 60-80: Ethyl acetate, 95:5) affording intermediate as a pale-yellow oil (4.7 g, 25 mmol, 18%). ^{1}H-NMR (400 MHz, $CDCl_3$) δH= 3.90 (s, 3H), 6.64 (d, J= 8.8 Hz, 1H), 7.61 (dd, J=2.8 Hz, J= 8.8 Hz), 8.18 (d, J= 2.8 Hz, 1H); ^{13}C-NMR (100 MHz, $CDCl_3$) δC= 53.6, 111.6, 112.5, 140.9, 147.5, 162.8.

6-methoxypyridine-3-carbaldehyde (5) [3]

In a solution of 5-bromo-2-methoxypyridine (**4**) (4.7 g, 25 mmol) in dry diethyl ether (50 mL) and under inert atmosphere, n-Butyl Lithium (2.5 M in hexanes, 12 mL, 30 mmol) was added at -35°C, and stirred until the formation of a brown precipitate. Then, dry N, N-dimethyl formamide (5.4 mL) was added dropwise for 5 min. The resulting mixture was stirred at 0°C (~2 h), protected from moisture and under inert atmosphere. Upon completion of the reaction, as it was indicated by TLC (SiO2, eluent Petroleum Ether 60:80: ethyl acetate, 80:20; UV light), the reaction was quenched by aqueous solution of ammonium chloride (5%, 25 mL). The aqueous layer was extracted with dichloromethane (3 x 50 mL). The combined organic extracts were dried over magnesium sulphate and concentrated under reduced pressure, forming the crude product as orange oil. The crude product was purified by automated flash chromatography column (Biotage IsoleraOne^{TM}); Rf = 0.43 (Petrol Ether 60-80: diethyl ether, 60:40) affording the product as yellow crystals (2.53 g, 16.7 mmol, 67%). Mp: 42-44°C [lit: 42-46°C]. ^{1}H-NMR (400 MHz, $CDCl_3$,) δH= 4.03 (s, 3H), 6.84 (d, J= 8.4 Hz, 1H), 8.05 (dd, J= 2.4 Hz, J= 8.4 Hz, 1H), 8.63 (d, J= 2.4 Hz, 1H), 9.96 (s, 1H); ^{13}C-NMR (100 MHz, $CDCl_3$) δC= 54.4, 112.2, 126.7, 137.5, 153.5, 167.8, 189.6.

(4Z)-4-[(6-methoxypyridin-3-yl)methylidene]-2-methyl-4,5-dihydro-1,3-oxazol-5-one (6) [4]

In a solution of 6-methoxypyridine-3-carbaldehyde (**5**) (1.84 g, 12.17 mmol) in acetic anhydride (8 mL), N-acetyl glycine (2.04 g, 17.44 mmol) and sodium acetate (1.5 g, 18.29 mmol) were added sequentially. The resulting mixture was stirred at 125°C for 4 h. Upon completion of the reaction, the mixture was poured into ice-water and stirred for a further 1 h leading to the formation of a yellow solid of the crude product which was collected by vacuum filtration, washed with water and dried in air. The crude product was purified by recrystallization from methanol affording the pure product as pale-yellow solid (1.66 g, 7.6 mmol, 63%). Mp: 152-154°C. 1H-NMR (400 MHz, $CDCl_3$) δH= 2.40 (s, 3H), 4.00 (s, 3H), 6.83 (d, J= 8.8 Hz, 1H), 7.12 (s, 1H), 8.6 (d, J= 2.4 Hz), 8.67 (dd, J= 2.4 Hz, J= 8.8 Hz); ^{13}C-NMR (100 MHz, $CDCl_3$) δC= 15.8, 54.1, 111.8, 123.3, 128.1, 132.0, 140.9, 152.1, 165.4, 165.7, 167.7. HRMS (ESI) for $C_{11}H_{10}N_2O_3$: theoretical [M+H]: 219.0691. measured [M+H]: 219.0766.

(2Z)-2-acetamido-3-(6-methoxypyridin-3-yl)prop-2-enoic acid (7) [4]

A solution of (4Z)-4-[(6-methoxypyridin-3-yl)methylidene]-2-methyl-4,5-dihydro-1,3-oxazol-5-one (**6**) (2 g, 9.16 mmol) in a mixture of water (30 mL)/acetone (50 mL) was refluxed for 9 h. The solution was allowed to cool down to room temperature and then was concentrated, under reduced pressure, forming the crude product as yellow solid. The crude product was purified by recrystallization from

methanol affording the product as pale brown crystals (1.76 g, 7.45 mmol, 81%). Mp: 162-164°C. ^{1}H-NMR (400 MHz, DMSO-d$_6$) δH= 2.00 (s, 3H), 3.89 (s, 3H), 6.88 (d, J= 8.8 Hz, 1H), 7.25 (s, 1H), 7.98 (dd, J= 2.4 Hz, J= 8.8 Hz, 1H), 8.40 (d, J= 2.4 Hz, 1H), 9.48 (s, 1H); ^{13}C-NMR (100 MHz, DMSO-d$_6$) δC= 23.2, 54.0, 111.1, 124.1, 127.1, 128.7, 139.8, 149.7, 164.1, 166.8, 169.7. HRMS (ESI) for $C_{11}H_{12}N_2O_4$: theoretical [M+H]: 237.0797, measured [M+H]: 237.0871.

2-acetamido-3-(6-methoxypyridin-3-yl)propanoic acid (8)

In a suspension of (2Z)-2-acetamido-3-(6-methoxypyridin-3-yl)prop-2-enoic acid (**7**) (1.76 g, 7.45 mmol) in methanol (50 mL), Pd/C (10%) was added. The reaction mixture was stirred under hydrogen gas at room temperature for 9 h. Upon completion of the reaction, the solution mixture was filtrated over a pad of Celite and then was concentrated under reduced pressure. The resulting slurry was purified by automated flash chromatography column (Biotage IsoleraOne^{TM}) (SiO$_2$); Rf = 0.89 (dichloromethane: methanol 9:1) affording the product as a pale-yellow oil which solidified on standing (940 mg, 3.94 mmol, 53%). Mp: 181-183°C. ^{1}H-NMR (400 MHz, CD$_3$OD) δH= 2.01 (3H, s), 2.95-2.30 (1H, m), 3.20-3.25 (m, 1H), 3.95 (s, 3H), 4.70-4.73 (m, 1H), 6.83 (d, J= 8.4 Hz, 1H), 7.67 (dd, J= 2.4 Hz, J= 8.4 Hz, 1H), 8.15 (d, J= 2.4 Hz, 1H); ^{13}C-NMR (100 MHz, CD$_3$OD) δC= 21.6, 34.1, 53.4, 54.1, 110.7, 126.4, 140.7, 147.2, 164.0, 172.5, 173.7. HRMS (ESI) for $C_{11}H_{14}N_2O_4$: theoretical [M+H]: 238.0953, measured [M+H]: 238.0961.

rac-5-(2-carboxy-2-acetamidoethyl)-2-methoxypyridin-1-ium-1-olate (9)

In a suspension of 2-acetamido-3-(6-methoxypyridin-3-yl)propanoic acid (**8**) (560 mg, 2.35 mmol) in a solution mixture of dichloromethane/methanol (9:1, 30mL) a sample of m-chloroperoxybenzoic acid (1 g, 5.8 mmol) was added. The resulting mixture was stirred at room temperature for 48 h under nitrogen atmosphere. Upon completion of the reaction, the solvents were carefully removed under reduced pressure (WARNING: Potential risk of explosion, use a blast shield) and the resulting yellowish slurry residue was washed several times with diethyl ether. Filtration of the product leaded to isolation of the title compound as a white powder (460 mg, 1.80 mmol, 77%). Mp: 189-192°C. ^{1}H-NMR (400 MHz, DMSO-d$_6$) δH= 1.79 (s, 3H), 2.71-2.77 (m 1H), 2.95-2.99 (m, 1H), 3.93 (s, 3H), 4.38-4.43 (m, 1H), 7.13 (d, J= 8.7 Hz, 1H), 7.23 (dd, J= 2.0 Hz, J= 8.7 Hz, 1H), 8.11 (d, J= 2.0 Hz, 1H), 8.23 (1H, d, J=8.7 Hz); ^{13}C-NMR (100 MHz, DMSO-d$_6$) δC= 22.8, 33.2, 53.2, 57.5, 109.0, 128.1, 128.3, 139.9, 157.5, 169.8, 173.2. HRMS (ESI) for $C_{11}H_{14}N_2O_5$: theoretical [M+H]: 254.0902, measured [M+H]: 254.0912.

2-amino-3-(1-hydroxy-6-oxo-1,6-dihydropyridin-3-yl)propanoic acid (SK-3)

A sample of 5-(2-carboxy-2-acetamidoethyl)-2-methoxypyridin-1-ium-1-olate (9) (460 mg, 1.80 mmol) was dissolved in concentrated solution of HCl (20 mL). The resulting mixture was refluxed for 3 h. Upon completion of the reaction, the mixture was concentrated to dryness leading to the formation of brownish crystals the target molecule (300 mg, 1.51 mmol, 84%). Mp: 120-122°C. ^{1}H-NMR (400 MHz, D$_2$O/CF$_3$COOD 8:2) δH= 2.96-3.10 (2H, m), 4.13 (d, J= 4.8 Hz, 1H), 6.63 (t, J= 9.2 Hz, 1H), 7.43 (d, J= 9.2 Hz, 1H), 7.94 (s, 1H), 8.49 (s, 3H); ^{13}C-NMR (100 MHz, D$_2$O/CF$_3$COOD 8:2) δC= 31.4, 52.9, 118.9, 136.6, 140.7, 140.8, 157.8, 170.4. For analytical purpose, a small portion of the titled molecule was dissolved in water and basified (pH 5.0) with ammonium hydroxide solution and kept at 5°C for several weeks until precipitation. HRMS (ESI) for $C_8H_{10}N_2O_4$: theoretical [M+H]: 199.0713, measured [M+H]: 199.0711.

Synthesis of SK5:

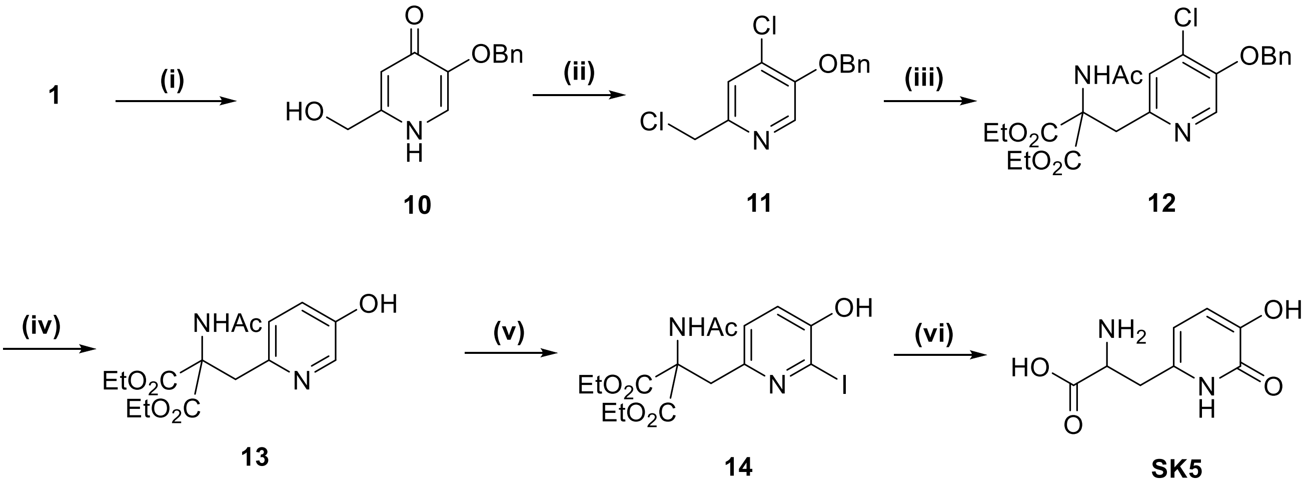

Fig. S4: Synthesis of SK5: Reagents and conditions: (**i**) conc. NH_4OH, 5 h, 120°C, (**ii**) $POCl_3$, 40 min, 120°C, (**iii**) Diethyl acetamidomalonate, NaH (60% in mineral oil), $DMF_{(dry)}$, overnight, RT, (**iv**) H_2, 5% Pd/C, methanol, RT, (**v**) $Na_2CO_{3\,(aq)}$, I_2, KI, overnight, RT, (**vi**) **a**) $Ba(OH)_{2\,(aq)}$, 24 h, 120°C, 24 h **b**) conc. HCl, 180°C, 1 h, **c**) conc. NH_4OH, pH 5, 5°C.

5-benzyloxy-2-(hydroxymethyl)-1,4-dihydropyridin-4-one (10) [5]

In a stainless-steel pressure vessel, concentrated ammonium hydroxide (40 mL) was mixed with a sample of 5-benzyloxy-2-(hydroxymethyl)-4H-pyran-4-one (**1**) (25 g, 107.64 mmol). The resulting mixture was heated at 120°C for 5 h. Upon completion of the reaction, volatiles were removed under reduced pressure. The resulting slurry was extracted with hot acetone, filtrated and washed with excess of hot acetone affording the titled compound as brown crystal (20 g, 86.48 mmol, 80%). Mp: 228-232°C [lit. 230-235°C]. ^{1}H-NMR (400 MHz, DMSO-d_6) δH= 4.34 (s, 2H), 5.00 (s, 2H), 6.23 (s, 1H), 7.25-7.35 (m, 6H); ^{13}C-NMR (100 MHz, DMSO-d_6) δC= 60.3, 70.9, 112.0, 124.0, 128.2, 128.3, 128.4, 128.7, 128.8, 137.8, 147.0, 149.7, 171.6.

5-benzyloxy-4-chloro-2-(chloromethyl)pyridine (11) [5]

A sample of 5-benzyloxy-2-(hydroxymethyl)-1,4-dihydropyridin-4-one (**10**) (13.84 g, 60 mmol) was added to a suspension of phosphorus oxychloride (42 mL), in portions, thus increasing the temperature of the reaction. After the solution mixture was returned back to room temperature, it was heated at 120°C for 40 min. Upon completion of the reaction, the mixture was poured into ice-water and stirred vigorously. Addition of more ice into the stirred mixture enhanced the hydrolysis of phosphorous oxychloride and led to the precipitation of the pure product (14.5 g, 60 mmol, 87%) as a black solid which was isolated by filtration and left to dry overnight. Mp: 77-79°C [lit: 80-81°C]. ^{1}H-NMR (400 MHz, DMSO-d_6) δH= 4.74 (s, 2H), 5.38 (s, 2H), 7.36-7.50 (m, 5H), 7.76 (s, 1H), 8.53 (s, 1H); ^{13}C-NMR (100 MHz, DMSO-d_6) δC= 45.4, 71.5, 125.7, 128.2, 128.4, 128.8, 129.1, 133.1, 135.5, 136.2, 149.4, 150.8.

1,3-diethyl-2-([5-benzyloxy-4-chloropyridin-2-yl]methyl)-2-acetamidopropanedioate (12) [5]

In dry N,N- dimethylformamide (62 mL) sodium hydride (60% in mineral oil, 2.16 g, 90 mmol) was added. The solution was stirred at room temperature and then diethyl acetamidomalonate (11.25 g, 51.8 mmol) was added in portion evolving hydrogen gas. Upon ceasing of hydrogen gas evolution, 5-benzyloxy-4-chloro-2-(chloromethyl)pyridine (**11**) (13.84 g, 51.6 mmol) was added. The resulting solution mixture was stirred overnight at room temperature. Upon completion of the reaction, acetic acid (25 mL) was added to neutralise the reaction mixture which was then concentrated, under reduced pressure, and the resulting syrup was dissolved in water (200mL) and extracted with diethyl ether (2 x 80 mL). The combined organic extracts were washed with brine, dried over magnesium sulphate and concentrated under reduced pressure, affording the titled compound as white crystals (22.7 g, 50.6 mmol, 98%), which was recrystallized from ethanol. Mp: 119-122°C [Lit: 118-120°C]. ^{1}H-NMR (400 MHz, DMSO-d_6) δH= 1.18 (t, J= 7.2 Hz, 6H), 1.82 (s, 3H), 3.50 (s, 2H), 4.08 (q, J= 7.2 Hz, J= 14.4 Hz, 4H), 5.25 (s, 2H), 7.13 (1H, s), 7.30-7.44 (m, 5H), 8.02 (s, 1H), 8.34 (s, 1H); ^{13}C-NMR (100 MHz, DMSO-d_6) δC= 14.3, 22.5, 40.1, 62.3, 66.6, 71.1, 125.8, 128.2, 128.7, 129.1, 131.3, 136.0, 136.6, 149.6, 149.9, 167.5, 169.9.

1,3-diethyl 2-acetamido-2-[(5-hydroxypyridin-2-yl)methyl]propanedioate (13) [5]

A suspension of diethyl 2-([5-benzyloxy-4-chloropyridin-2-yl]methyl)-2-acetamidopropanedioate (**12**) (6 g, 13.36 mmol) in methanol (75 mL), sodium acetate (6 g, 73.14 mmol) and a catalytic amount of 10% Pd/C was stirred vigorously under hydrogen atmosphere. Upon completion of the reaction, as indicated by TLC (SiO_2, ethyl acetate 100%), the solution mixture was filtered over a pad of Celite, washed with methanol and diluted with water (150 mL) forming the titled compound as white crystals (2.94 g, 9.06 mmol, 68%) Mp: 152-154°C [Lit: 150-153°C]. ^{1}H-NMR (400 MHz, DMSO-d_6) δH= 1.1 (t, J= 7.2 Hz, 6H), 1.81 (s, 3H), 3.43 (s, 2H), 4.07 (q, J= 7.2 Hz, 4H), 6.78 (d, J= 8.8 Hz, 1H), 6.99 (dd, J= 3.2 Hz, J= 8.8 Hz, 1H), 7.87 (s, 1H), 7.91 (d, J= 3.2 Hz, 1H); ^{13}C-NMR (100 MHz, DMSO-d_6) δC= 14.3, 22.6, 39.9, 66.8, 69.0, 122.8, 125.1, 137.6, 146.5, 152.8, 167.7, 169.7.

1,3-diethyl-2-acetamido-2-[(5-hydroxy-6-iodopyridin-2-yl)methyl]propanedioate (14) [5]

A sample of diethyl 2-acetamido-2-[(5-hydroxypyridin-2-yl)methyl]propanedioate (**13**) (2.5 g, 7.7 mmol) was dissolved in water (70 mL) containing sodium carbonate (1.54 g, 13.97 mmol). A solution of iodine (1.92 g, 15.13 mmol) and potassium iodide (2.32 g, 13.97 mmol) in water (50 mL) was added dropwise to the previous solution. The resulting mixture was stirred overnight at room temperature. Upon completion of the reaction, the solution mixture was neutralized with glacial acetic acid (5 mL), leading to the precipitation of the titled compound which was collected by filtration, washed with water and dried at 90°C. The titled compound was obtained as a white powder (3.39 g, 7.54 mmol, 98%) Mp: 200-203°C [Lit: 196-197°C]. ^{1}H-NMR (400 MHz, DMSO-d_6) δH= 1.15 (t, J= 7.2 Hz, 6H), 1.83 (s, 3H), 3.34 (s, 2H), 4.11 (q, J= 7.2 Hz, 4H), 6.78 (d, J= 8 Hz, 1H), 6.98 (d, J= 8 Hz, 1H), 7.9 (s, 1H); ^{13}C-NMR (100 MHz, DMSO-d_6) δC= 14.4, 22.5, 39.0, 62.2, 66.8, 111.1, 121.7, 125.1, 148.0, 153.2, 167.5, 169.9.

2-amino-3-(5-hydroxy-6-oxo-1,6-dihydropyridin-2-yl)propanoic acid (SK-5) [5]

A mixture of barium hydroxide (7.1 g, 41.43 mmol) and diethyl 2-acetamido-2-[(5-hydroxy-6-iodopyridin-2-yl)methyl] propanedioate (**14**) (4 g, 8.88 mmol) in water (70 mL) was refluxed for 24 h. Upon completion of the reaction, the resulting barium salt was collected and refluxed with concentrated HCl (50 mL) for 1 h. Once again, upon completion of the reaction, the solution mixture was evaporated to dryness yielding a yellowish salt of the crude product which was dissolved in water

(20 mL), treated with charcoal and filtered. The pH of the filtrates was adjusted to 5.0 by addition of concentrated ammonia leading to precipitation of the titled compound appearing as white crystals (1.67 g, 8.44 mmol, 95%) Mp: 193-195°C [Lit: 196-197°C]. ^{1}H-NMR (400 MHz, DMSO-d_6) δH= 2.86-2.99 (m, 2H), 4.07 (t, J= 7.6 Hz, 1H), 6.06 (d, J= 7.2 Hz, 1H), 6.75 (d, J= 7.2 Hz, 1H); ^{13}C-NMR (100 MHz, DMSO-d_6) δC= 32.4, 52.0, 110.8, 121.2, 131.3, 144.7, 159.0, 170.3. HRMS (ESI) for $C_9H_{10}N_2O_4$: theoretical [M+H]: 213.0869, measured [M+H]: 213.0866.

Synthesis of SK4C1 and SK4C2:

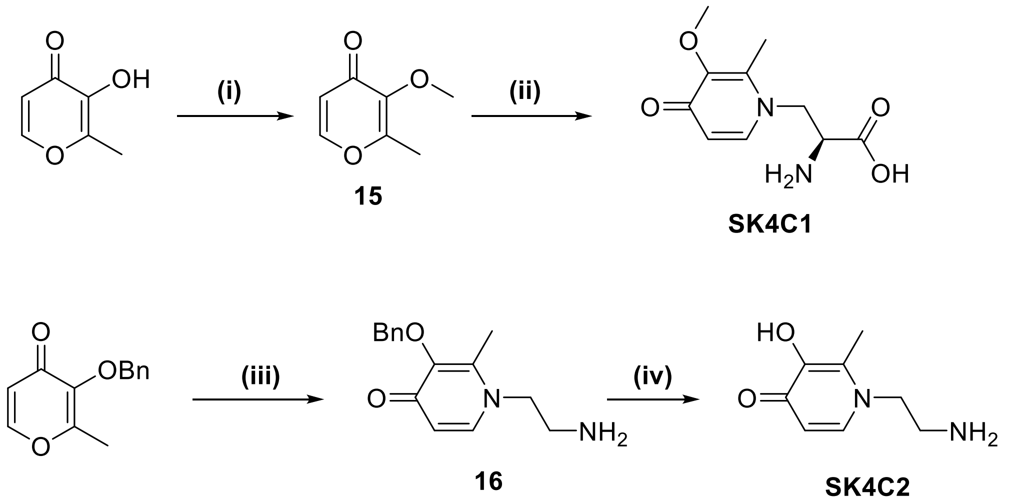

Fig. S5:Synthesis of SK4C1 and SK4C2: Reagents and conditions: (**i**) MeI, K_2CO_3, acetone, 3 h, reflux, 94% ; (ii) a) (2S)-3-amino-2-([(tert-butoxy)carbonyl]amino)propanoic acid, NaOH, RT, 8 days, b) HBr, reflux, 20 min, c) NH_3, pH 5, 5°C, 72 h, 42%; (iii) Ethylenediamine, NaOH, MeOH, H_2O, reflux, 4 h, 77%; (iv): H_2, Pd/ C (10%), MeOH, RT, 3 h, 95%.

3-methoxy-2-methyl-4H-pyran-4-one (15) [6]

In a suspension of maltol (5 g, 41.28 mmol) in acetone (50 mL) potassium carbonate (16.4 g, 118.66 mmol) and iodomethane (7.4 g, 49.31 mmol) were added sequentially. The resulting solution mixture was refluxed for 3 h. Upon completion of the reaction, the solvents were removed under reduced pressure and the resulting residue was partitioned in a mixture of dichloromethane/water (1:1) (100 mL). Then the aqueous phase was extracted with dichloromethane (3x50 mL). The combined organic extracts were washed with brine, dried over magnesium sulphate and concentrated under reduced pressure to afford compound 15 as a brown-orange oil (5.44 g, 38.85 mmol, 94%). ^{1}H-NMR (400 MHz, $CDCl_3$) δH: 2.35 (s, 3H), 3.85 (s, 3H), 6.35 (d, J= 10.8 Hz, 1H), 7.68(s, J= 10.8 Hz, 1H); ^{13}C-NMR (100 MHz, $CDCl_3$) δC: 14.3, 59.8, 117.0, 145.3, 153.7, 159.1, 175.1.

Synthesis of 2-amino-3-(3-methoxy-2-methyl-4-oxo-1,4-dihydropyridin-1-yl)propanoic acid (SK4C1)

In a suspension of 3-methoxy-2-methyl-4H-pyran-4-one (**15**) (5.44 g, 38.85 mmol) in water (145 mL) and ethanol (73 mL) containing sodium hydroxide (1.5 g, 37.5 mmol), a sample of 3-amino-2-([(tert-butoxy)carbonyl]amino)propanoic acid (5.10 g, 25.0 mmol) was added. The resulting solution mixture was stirred at room temperature for 7 days. Upon completion of the reaction, the pH was adjusted to 2 by the addition of conc. HCl and the solvents were evaporated to dryness under reduced pressure. The resulting residue was refluxed with hydrobromic acid (30% w/v, 20 mL) for 20 min. Then the

solvents were evaporated and the residue was dissolved in water (30 mL), treated with charcoal and filtered. Addition of conc. ammonium hydroxide solution to the filtrates until pH 5 cause the crystallisation of the titled product which was collected and by filtration as a brown solid (2.37 g, 10.5 mmol, 42%). Mp: 170-173°C. ^{1}H-NMR (400 MHz, D_2O/CF_3COOD, 8:2) δH: 2.13 (s, 3H), 3.27 (s, 3H), 4.09 (t, J= 7.2 Hz, 1H), 4.28-4.51 (m, 2H), 6.7 (d, J= 7.2 Hz, 1H), 7.76 (d, J= 7.2 Hz, 1H); ^{13}C-NMR (100 MHz, 400 MHz, D_2O/CF_3COOD, 8:2) δC: 12.8, 49.3, 51.1, 60.9, 119.5, 142.9, 144.7, 150.7, 167.5, 176.3. HRMS (ESI) for $C_{10}H_{14}N_2O_4$: theoretical [M+H]: 227.1032, measured [M+H]: 227.1022.

Synthesis of 1-(2-aminoethyl)-3-benzyloxy-2-methyl-1,4-dihydropyridin-4-one (16)

In a suspension of 3-benzyloxy-2-methyl-1,4-dihydropyridin-4-one (3 g, 13.9 mmol) in EtOH: water mixture (20:15 mL) sodium hydroxide (0.5 g, 12.5 mmol) was added. Ethylenediamine (3.3 mL, 49.3 mmol) was added to the stirring solution mixture and the resulting solution was heated at 70°C for 4 h and then it was allowed to cool to room temperature overnight. The solution mixture was acidified to pH 1 by the addition of concentrated hydrochloric acid. The resulting mixture was concentrated under reduced pressure affording an orange salt which was washed with excess of acetone. The salt was collected by filtration, dissolved in water (20 mL), basified to pH 12 by aqueous solution of sodium hydroxide (10 M) and extracted in dichloromethane (4x50 mL). The organic extracted were concentrated under reduced pressure affording the title compound as brown oil (2.79 g, 10.8 mmol, 77%). ^{1}H-NMR (400 MHz, $CDCl_3$) δH: 2.15 (s, 3H), 2.67 (t, J= 7.6 Hz, 2H), 4.23 (t, J= 7.6 Hz, 2H), 5.18 (s, 2H), 6.57 (d, J= 6.8 Hz, 1H), 7.31-7.38 (m, 5H), 7.61 (d, J= 6.8 Hz, 1H); ^{13}C-NMR (100 MHz, $CDCl_3$) δC:13.5, 34.0, 52.2, 75.0, 111.2, 114.1, 114.2, 113.9, 128.9, 158.3, 158.7, 159.1, 159.5.

Synthesis of 1-(2-aminoethyl)-3-hydroxy-2-methyl-1,4-dihydropyridin-4-one (SK4C2)

In a suspension of 1-(2-aminoethyl)-3-(benzyloxy)-2-methyl-1,4-dihydropyridin-4-one (**16**) (2 g, 7.74 mmol) in methanol (30 mL) a catalytic amount of Pd/C (10%, 580 mg, 5.45 mmol) was added. The reaction mixture was stirred under hydrogen gas at room temperature for 3 h. Upon completion of the reaction, the solution mixture was filtrated over a pad of Celite, and concentrated under reduced pressure affording the title compound as pale yellow crystals (1.17 g, 6.97 mmol, 90%). Mp: 143-148°C. ^{1}H-NMR (400 MHz, DMSO-d_6) δH: 2.56 (s, 3H), 2.86 (t, J= 6.8 Hz, 2H), 4.52 (t, J= 6.8 Hz, 2H), 7.37 (d, J= 6.8 Hz, 1H), 8.28 (d, J= 6.8 Hz, 1H); ^{13}C-NMR (100 MHz, DMSO-d_6) δC: 13.0, 34.1, 52.1, 111.0, 138.9, 142.4, 143.2, 159.0. HRMS (ESI) for $C_8H_{12}N_2O_4$: theoretical [M+H]: 169.0977, measured [M+H]: 169.0968.

Supplementary Figures

Fig. S6A

	SK2	SK3	SK4	DFP[a]	1,2-HOPO[a]
**LH**	8.84(1)	8.99(2)	9.82(9)	9.9	5.8
**LH_2**	7.48(1)	5.59(1)	7.0(3)	3.6	/
**LH_3**	4.72(1)	2.0(1)	2.82(6)	/	/
**LH_4**	<-0.5	~-0.2	<-1	/	/

Solvent: H_2O, I = 0.1 M ($NaClO_4$), T = 25.0 °C. The numbers given in parentheses correspond to the SD expressed to the last significant figure. [a] Liu & Hider, 2002

Fig. S6B

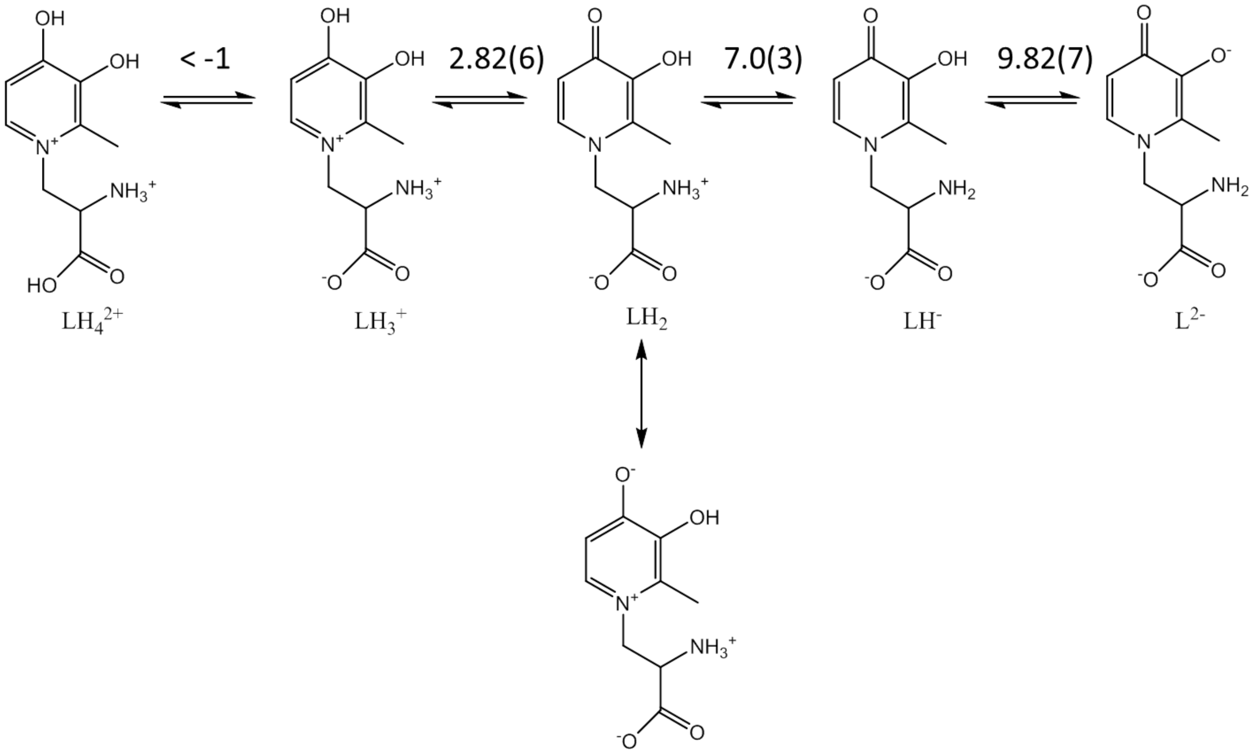

Fig. S6C

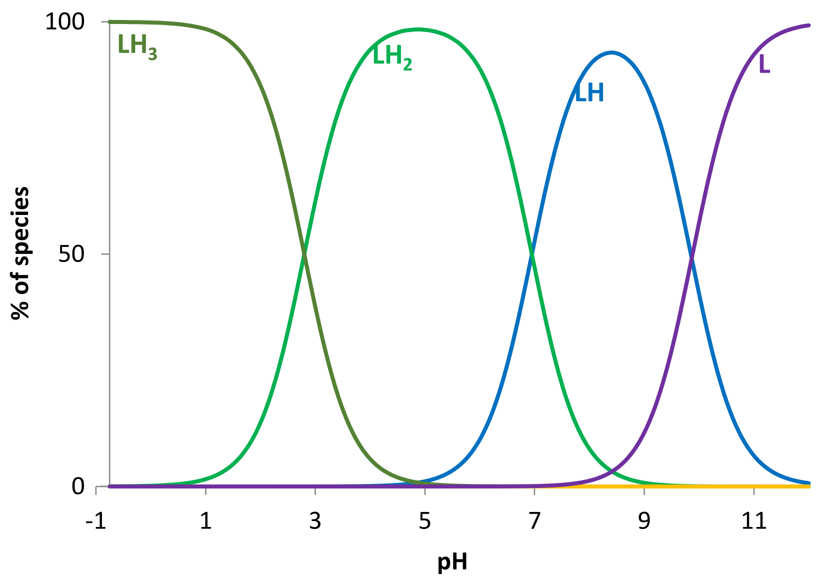

Fig. S6: Overview of the protonation constants of ligands

A. Protonation constants (pKa) of ligands SK2, SK3, SK4, 1,2-HOPO and DFP. **B** protonation scheme of SK4 and **C**. Distribution curves of SK4 indicating the different structures available in solution as a function of pH . As LAT1 is known to accept large neutral amino acids (in their zwitterionic form), only species LH_2 is expected to be a substrate. It represents 25% of SK4 species at pH 7.4.

Fig. S7A:

	SK-2	SK-3	SK-4	1,2-HOPO[a]	DFP[b]	3,4-HOPO
MLH_2	22.9					
MLH		21.83(1)	21.58(1)			
ML				10.6	15.0	14.26(3)
ML_2H_2		39.06(1)	40.65(1)			
ML_2				19.3	27.3	25.73(1)
ML_3H_3		53.89(1)	58.87(6)			
ML_3H_2						
ML_3			40.62(7)	27.2	37.43	34.91(1)
$ML_2(OH)_2$			13.54(2)			

Solvent: H_2O, I = 0.1 M ($NaClO_4$), T = 25.0 °C. The numbers given in parentheses correspond to the SD expressed to the last significant figure. charges were omitted for the sake of clarity. [a] Li & Martell, 1993. [b] Nurchi et al. 2008. log β_{MLxHy} values represent the overall stability constants of the following equilibria :

$Fe + L_x + H_y \overset{\beta_{MLxHy}}{\rightleftarrows} ML_xH_y$,where $\beta_{MLxHy} = [ML_xH_y]/[M][L]_x[H]_y$.

Fig. S7B:

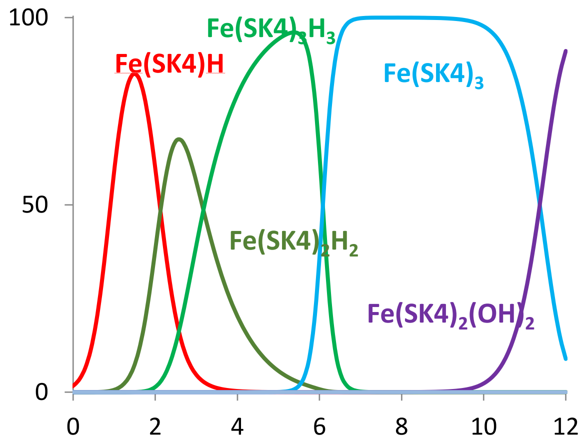

Fig. S7: Iron chelation properties of ligands SK2, SK3, SK4 and relevant ligands frome literature

A. Stability constants (log β_{MLxHy}) of the Fe(III) complexes of ligands SK2, SK3, SK4, 3,4-HOPO and DFP. **B.** Distribution curves (% of species versus pH) of SK4 Fe(III) complexes. It can be observed that SK4 is present as tris-bidentate Fe(III) complex over the relevant pH range (pH 5.5 to 8).

Fig. S8:

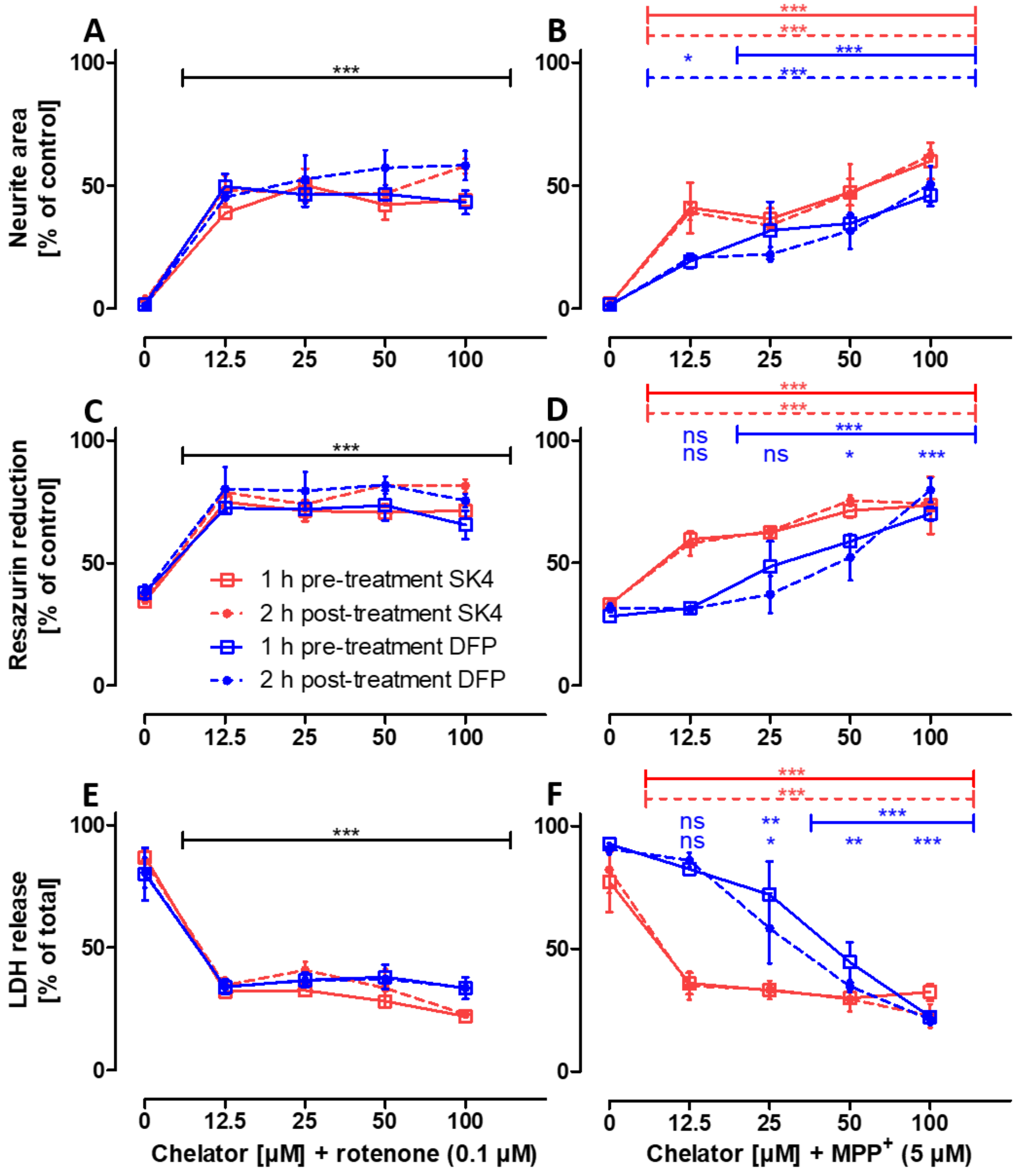

Fig. S8: Comparison of treatment schedules with iron chelators

Differentiated LUHMES neurons were treated on day 6 (d6) with rotenone (0.1 µM; **A/C/E**) or MPP^+ (5 µM; **B/D/F**) in the presence or absence of the iron chelators SK4 (red) or deferiprone (DFP; blue). The chelators were added 1 h prior (open squares) or 2 h after (filled circles) toxicant exposure, as indicated. The toxicant was incubated for 72 h. **A/B:** The cells were stained with calcein-AM (live cells) and H-33342 (nuclei) and neurite integrity was quantified by automated microscopy. **C/D:** Resazurin-reduction was assessed as viability endpoint. **E/F:** The release of lactate dehydrogenase (LDH) was measured as death endpoint. Data is presented as means ± SD of three replicates. Statistical analysis was performed with a one-way ANOVA followed by Dunnet‘s post test *(P < 0.05), **(P < 0.01), ***(P < 0.001).

Supplementary references

Fredenburg AM, Sethi RK, Allen DD, & Yokel RA (1996). The pharmacokinetics and blood-brain barrier permeation of the chelators 1,2 dimethly-, 1,2 diethyl-, and 1-[ethan-1'ol]-2-methyl-3-hydroxypyridin-4-one in the rat. Toxicology 108**:** 191-199.

Harris R (1976). Potential wool growth inhibitors. Improved syntheses of mimosine and related 4(1H

Harris R, & Teitei T (1977). Potential wool growth inhibitors. 2(1H)-Pyridone analogues of mimosine. Australian Journal of Chemistry 30**:** 649-655.

Kawasuji T, Johns BA, Yoshida H, Weatherhead JG, Akiyama T, Taishi T*, et al.* (2013). Carbamoyl pyridone HIV-1 integrase inhibitors. 2. Bi- and tricyclic derivatives result in superior antiviral and pharmacokinetic profiles. J Med Chem 56**:** 1124-1135.

Kiss LE, & Soares-da-Silva P (2014). Medicinal chemistry of catechol O-methyltransferase (COMT) inhibitors and their therapeutic utility. J Med Chem 57**:** 8692-8717.

Li YJ, & Martell AE (1993) Potentiometric and spectrophotometric determination of stabilities of the 1-hydroxy-2-pyridinone complexes of trivalent and divalent metal ions. Inorg. Chim. Acta 214: 103-111.

Liu ZD, & Hider RC (2002). Design of iron chelators with therapeutic application. Coordination Chemistry Reviews 232**:** 151-171.

Nurchi VM, Crisponi G, Pivetta T, Donatoni M, & Remelli M (2008) Potentiometric, spectrophotometric and calorimetric study on iron(III) and copper(II) complexes with 1,2-dimethyl-3-hydroxy-4-pyridinone. J. Inorg. Biochem. 102: 684–692.

Radić Stojković M, Piotrowski P, Schmuck C, & Piantanida I (2015). A short, rigid linker between pyrene and guanidiniocarbonyl-pyrrole induced a new set of spectroscopic responses to the ds-DNA secondary structure. Organic & Biomolecular Chemistry 13**:** 1629-1633.

Roy S, Preston JE, Hider RC, & Ma YM (2010). Glucosylated deferiprone and its brain uptake: implications for developing glucosylated hydroxypyridinone analogues intended to cross the blood-brain barrier. J Med Chem 53**:** 5886-5889.

Scott LE, Telpoukhovskaia M, Rodriguez-Rodriguez C, Merkel M, Bowen ML, Page BDG*, et al.* (2011). N-Aryl-substituted 3-(beta-D-glucopyranosyloxy)-2-methyl-4(1H)-pyridinones as agents for Alzheimer's therapy. Chem Sci 2**:** 642-648.

Singh S, Epemolu RO, Dobbin PS, Tilbrook GS, Ellis BL, Damani LA*, et al.* (1992). Urinary metabolic profiles in human and rat of 1,2-dimethyl- and 1,2-diethyl-substituted 3-hydroxypyridin-4-ones. Drug Metab Dispos 20**:** 256-261.

Zheng H, Youdim MB, Weiner LM, & Fridkin M (2005). Novel potential neuroprotective agents with both iron chelating and amino acid-based derivatives targeting central nervous system neurons. Biochem Pharmacol 70**:** 1642-1652.
